# EEG-based sensorimotor neurofeedback for motor neurorehabilitation in children and adults: A scoping review

**DOI:** 10.1016/j.clinph.2024.08.009

**Published:** 2024-11

**Authors:** Elena Cioffi, Anna Hutber, Rob Molloy, Sarah Murden, Aaron Yurkewich, Adam Kirton, Jean-Pierre Lin, Hortensia Gimeno, Verity M. McClelland

**Affiliations:** a Department of Clinical Neuroscience, Institute of Psychiatry, Psychology and Neuroscience, King’s College London, London, UK; b Department of Paediatric Neurosciences, Evelina London Children’s Hospital, London, UK; c Islington Paediatric Occupational Therapy, Whittington Hospital NHS Trust, London, UK; d Barts Bone and Joint Health, Blizard Institute, Queen Mary University of London, London, UK; e Department of Paediatric Neurology, King’s College Hospital NHS Foundation Trust, London, UK; f Mechatronics Engineering, Ontario Tech University, Ontario, Canada; g Department of Clinical Neurosciences, Cumming School of Medicine, University of Calgary, Calgary, AB, Canada; h The Royal London Hospital and Tower Hamlets Community Children’s Therapy Services, Barts Health NHS Trust, London, UK

**Keywords:** Movement Disorders, Neurofeedback, Brain-Computer Interface, EEG, Neurorehabilitation, Children

## Abstract

**Objective:**

Therapeutic interventions for children and young people with dystonia and dystonic/dyskinetic cerebral palsy are limited. EEG-based neurofeedback is emerging as a neurorehabilitation tool. This scoping review maps research investigating EEG-based sensorimotor neurofeedback in adults and children with neurological motor impairments, including augmentative strategies.

**Methods:**

MEDLINE, CINAHL and Web of Science databases were searched up to 2023 for relevant studies. Study selection and data extraction were conducted independently by at least two reviewers.

**Results:**

Of 4380 identified studies, 133 were included, only three enrolling children. The most common diagnosis was adult-onset stroke (77%). Paradigms mostly involved upper limb motor imagery or motor attempt. Common neurofeedback modes included visual, haptic and/or electrical stimulation. EEG parameters varied widely and were often incompletely described. Two studies applied augmentative strategies. Outcome measures varied widely and included classification accuracy of the Brain-Computer Interface, degree of enhancement of mu rhythm modulation or other neurophysiological parameters, and clinical/motor outcome scores. Few studies investigated whether functional outcomes related specifically to the EEG-based neurofeedback.

**Conclusions:**

There is limited evidence exploring EEG-based sensorimotor neurofeedback in individuals with movement disorders, especially in children. Further clarity of neurophysiological parameters is required to develop optimal paradigms for evaluating sensorimotor neurofeedback.

**Significance:**

The expanding field of sensorimotor neurofeedback offers exciting potential as a non-invasive therapy. However, this needs to be balanced by robust study design and detailed methodological reporting to ensure reproducibility and validation that clinical improvements relate to induced neurophysiological changes.

## Highlights

• EEG-based sensorimotor neurofeedback is sparsely explored in children with motor disorders and adult populations beyond stroke.
• Most brain-computer interfaces use upper limb motor imagery to trigger visual, haptic or electrical stimulation neurofeedback.
• Reporting of EEG neurofeedback parameters and outcomes varies widely: greater transparency is required to validate brain-behaviour changes.

## 1 Introduction

Dystonia and dystonic/dyskinetic cerebral palsy (CP) are neurological movement disorders, characterised by sustained or intermittent muscle contractions resulting in abnormal, often painful, twisting movements and postures (Albanese et al., 2013). Whilst adult-onset dystonia is commonly localised and not progressive, childhood-onset dystonia is more often severe and generalised (Bressman, 2004). Dystonia and dystonic/dyskinetic CP are life-long conditions that adversely affect quality of life, causing physical and psychological challenges (Girach et al., 2019, Skogseid et al., 2007, Zurowski et al., 2013). There is no cure and management options are limited. Neuromodulation with Deep Brain Stimulation (DBS) of the globus pallidus internus has yielded considerable benefits in some individuals with severe, medically refractory dystonia (Gimeno et al., 2013, Marks et al., 2013, Romito et al., 2015, Vidailhet et al., 2009). However, this invasive technique presents its own set of challenges, such as risk of infection and patient anxiety surrounding surgery. Further, patient outcomes of DBS are variable: individuals with acquired dystonia (dystonic/dyskinetic CP) respond more modestly than those with genetic/idiopathic dystonia (Elkaim et al., 2019, Koy et al., 2013, Lumsden et al., 2022, Marks et al., 2013, Vidailhet et al., 2009) indicating a significant need for alternative therapies for this population.

Researchers have begun to explore the efficacy of other therapeutic strategies to augment the outcomes of DBS. For example, proof-of-concept for a rehabilitation intervention, the Cognitive Orientation to daily Occupational Performance (Polatajko & Mandich, 2004) (CO-OP) has been established for childhood-onset hyperkinetic movement disorders (Gimeno et al., 2019, Gimeno et al., 2021, Gimeno et al., 2020). CO-OP is a performance-based, client-centred intervention aimed at improving performance in self-identified functional goals, instead of solely focusing on reducing dystonic symptoms. Although this work is encouraging, non-pharmacological and non-invasive interventions are lacking, and there is a critical clinical need to develop innovative therapies. Further work to understand the pathophysiology underlying dystonia and dystonic/dyskinetic CP is key to informing the development of new data-driven therapeutic approaches.

Recent research using electroencephalography (EEG) demonstrates that cortical sensorimotor processing, specifically modulation of the brain rhythm ‘mu’, is abnormal in children and young people (henceforth referred to as children) with dystonia and dystonic/dyskinetic CP (McClelland et al., 2021, McClelland and Lin, 2021). Arising from the central/midline fronto-parietal sensorimotor brain region, the mu rhythm, also known as the sensorimotor rhythm (SMR), comprises two components, a prominent alpha/mu (8–13 Hz) rhythm and a smaller contribution from a beta (13–30 Hz) rhythm, which have a near harmonic relationship (Wischnewski et al., 2022). Mu is strongly associated with cortical sensorimotor processing: in particular, mu oscillatory activity is reduced in power in response to movement or somatosensory stimulation. This phenomenon is termed an event-related desynchronisation (ERD) (Neuper et al., 2003, Pfurtscheller et al., 2000) and is considered to reflect activation of the sensorimotor cortex. This is usually followed by an event-related synchronisation (ERS) whereby the cortex is deactivated, which is associated with motor control and inhibition, movement outcome and error processing (Pfurtscheller, 2001, Torrecillos et al., 2015). Importantly, mu ERD and ERS can also be evoked by observed or imagined movement, a phenomenon which is exploited in the development of brain-computer interfaces (BCIs) (Broetz et al., 2010, Jeunet et al., 2019).

EEG-based BCI systems acquire and detect changes in cortical activity with high temporal resolution and translate EEG signals into output commands in real-time, allowing the participant to control an external device (such as a switch or a remote-controlled wheelchair) or engage with computer systems. In addition to enabling device control, the closed-loop paradigm of a BCI can provide real-time neurofeedback of a specific brain rhythm via various modalities such as visual, auditory, haptic or electrical stimulation. The neurofeedback encourages the participant to gain voluntary control and self-regulation of the selected brain rhythm, usually the mu/SMR activity (Sitaram et al., 2017), through operant conditioning or associative learning, capitalising on the Hebbian-associated and long-term potentiation-like mechanisms of neuroplasticity (Mrachacz-Kersting et al., 2012, Sitaram et al., 2017). Thus, while some EEG-based BCI systems are developed as assistive devices for those with difficulties to communicate or perform motor activities, others are designed specifically for neurorehabilitation. The latter focus on the modulation of mu to enhance sensorimotor control, with the potential of improving motor capabilities and alleviating clinical symptoms (Young et al., 2021). Positive effects of neurofeedback have been demonstrated in adults with stroke (Broetz et al., 2010, Cervera et al., 2018, Jeunet et al., 2019, Remsik et al., 2019). However, the application of EEG-based BCIs as a neurorehabilitation technique in children (Kinney-Lang et al., 2016) and adults with dystonia or dystonic/dyskinetic CP is relatively unexplored.

Motor imagery (MI) has been used as a cognitive strategy by people with Parkinson’s Disease (Nonnekes et al., 2019), and also in children with hyperkinetic movement disorders including dystonia and dystonic/dyskinetic CP (Butchereit et al., 2022) who showed subsequent improvement in motor performance and skills acquisition (Gimeno et al., 2019, Gimeno et al., 2020). Other strategies include distraction, mental self-guidance, internally/externally focused attention, and emotional regulation (Butchereit et al., 2022). It is likely that the use of MI as a strategy engages mu modulation and activation of the sensorimotor network, relevant to many EEG-BCI systems, whilst other cognitive strategies such as mindfulness meditation can improve EEG-BCI control in healthy adults (Stieger et al., 2021, Tan et al., 2014). We were therefore interested in how such strategies have been used in EEG-neurofeedback studies in populations with neurological disorders.

### 1.1 Aim of study

We planned to conduct a scoping review to establish (i) the extent of research investigating EEG-based sensorimotor neurofeedback techniques in children with dystonia and dystonic/dyskinetic CP, and (ii) whether any (cognitive) strategies have been used to augment neurofeedback.

From an initial search of the literature, we found no evidence of EEG-based sensorimotor neurofeedback research in children with dystonia or dystonic/dyskinetic CP. Further, our search identified only two studies exploring such techniques in adults with dystonia. Expanding the search to include both adults and children with CP (all types, rather than specifically dystonic/dyskinetic) still only yielded six articles. Therefore, the scoping review was broadened to include adult and paediatric populations and to span a wider range of neurological motor impairments.

## 2 Methods

This study followed the Joanna Briggs Institute guidelines (Peters et al., 2020), underpinned by the Arksey and O’Malley (Arksey & O'Malley, 2005) and Levac and colleagues (Levac et al., 2010) frameworks, and was conducted in line with the Preferred Reporting Items for Systematic Reviews and Meta-Analyses extension for Scoping Reviews (PRISMA-ScR) Checklist (Tricco et al., 2018). The review protocol was registered prospectively in the Open Science Framework (OSF) database (DOI 10.17605/OSF.IO/SKH85).

### 2.1 Development of the research question

The research question was developed using the Population, Concept, Context framework (Peters et al., 2020) and extended as outlined in the aims above. The primary research question which guided the review was: ‘How has EEG-based sensorimotor neurofeedback been used in rehabilitation for children and adults with neurological motor impairments?’. The secondary question was, ‘Have other techniques been used to augment this feedback?’.

### 2.2 Search strategy

Two reviewers independently conducted an initial search in MEDLINE and Cumulative Index to Nursing and Allied Health Literature (CINAHL) databases to gain an understanding of the breadth of relevant literature. Text words in the titles and abstracts were used to establish search terms and develop the full search strategy (Supplementary Material, Appendix A), which was subsequently reviewed by a librarian following the Peer Review of Electronic Search Strategies (PRESS) checklist (McGowan et al., 2016). The final, full search strategy was used to identify literature published up until August 2022 in MEDLINE, CINAHL and Web of Science, with the search strategy modified for each database where necessary. Due to the high volume of papers and time taken to screen articles, a second more recent search with identical search parameters was conducted in October 2023 to ensure findings remained current and incorporated the most recently published research.

### 2.3 Study selection

The inclusion and exclusion criteria were developed and refined by the team, based on an initial screening of a small sample of articles. One modification was to exclude articles whereby feedback related only to electromyography (EMG), whereas articles using combined EMG and EEG feedback were retained.

The final inclusion criteria included: (1) patients with neurological motor impairments (i.e., stroke, dystonia, cerebral palsy, Parkinson’s Disease and multiple sclerosis); (2) Scalp EEG-BCI systems processing signals recorded over the sensorimotor cortex (encompasses mu, beta and alpha where this represented mu rhythms); (3) participants of all ages; (4) any type of experimental study designs reporting original data; (5) any publication year; (6) studies published in English. Although reviews were excluded, reference lists were examined to identify further eligible studies not already captured.

Titles and abstracts of identified articles were exported and uploaded into Rayyan screening software, and duplicates were removed. Titles and abstracts were screened by reviewers (EC, RM, SM) independently against the eligibility criteria, with at least 20% screened by at least two reviewers to ensure consistency. Any disputes were discussed until consensus was reached or resolved by a third reviewer. The full texts of included articles were examined further to confirm eligibility and reviewed independently by two reviewers (EC and VM), with at least 25% screened by both. Disagreements were resolved through discussion. Reasons for exclusion at full-text screening stage were documented, including those listed above and a further category for articles providing insufficient methodological information (e.g. EEG neurofeedback parameters unclear).

### 2.4 Data extraction

A data extraction form was included in the scoping review protocol and uploaded to OSF before commencing the study. Data extraction included study design, participant information (motor impairment diagnosis, age), sensorimotor task parameters, neurofeedback mode, EEG-based sensorimotor neurofeedback parameters, outcome measures and augmented strategy use.

Three members of the research team (EC, VM, HG) independently extracted and compared five articles of different methodologies to ensure data extraction captured all relevant aspects, resulting in some minor refinements. Data extraction was completed independently by two reviewers (EC and AH), with at least ten percent of included articles extracted by both reviewers to ensure consistency. Throughout the extraction process, team members met regularly to discuss any uncertainties and ensure accuracy. Disagreements between reviewers were resolved through discussion or by senior authors (VM and HG) when necessary.

## 3 Results

**Fig. 1:**
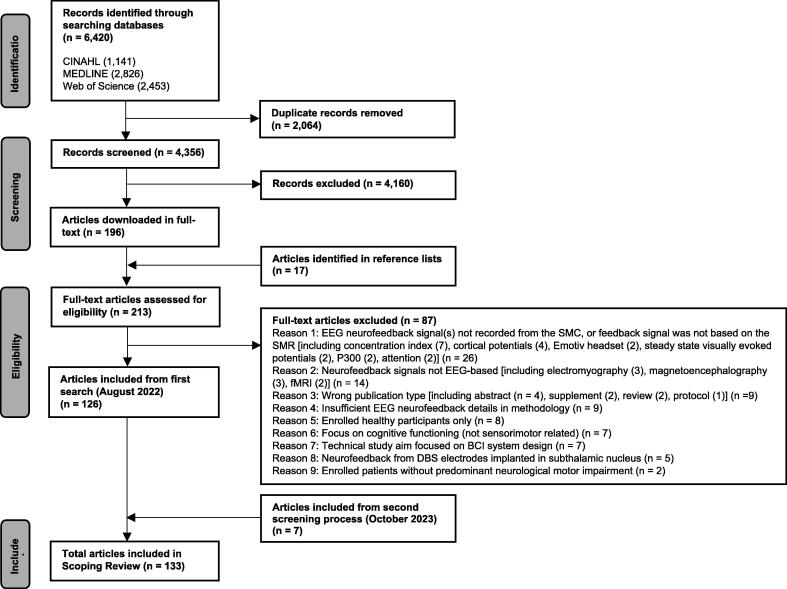
PRISMA flow chart of screening process for scoping review. BCI=Brain-Computer Interface, CINAHL=Cumulative Index to Nursing and Allied Health Literature, DBS=Deep Brain Stimulation, EEG=Electroencephalography, fMRI=Functional Magnetic Resonance Imaging, SMC=Sensorimotor Cortex, SMR=Sensorimotor Rhythm.

In the initial search, 126 articles out of 4,373 (total retrieved from database searching and reference lists after de-duplication) were included, based on the screening steps and exclusion reasons outlined in Fig. 1. A further seven articles were included from the second search. Thus, 133 articles were included in total. Most commonly, articles were excluded because the EEG-based neurofeedback signal(s) was not recorded from the sensorimotor cortex or, in some cases, this area was included in the recording, but the feedback signal was not based on the SMR (n = 26).

### 3.1 Year of publication

**Fig. 2:**
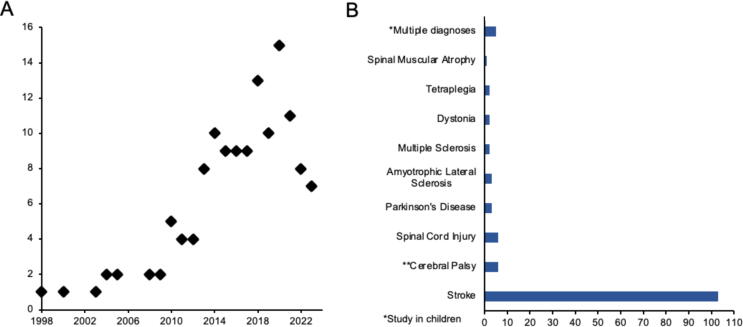
(A) Number of included articles published by year. (B) Neurological motor impairment diagnoses of study populations.

The 133 included articles are listed in Table 1. All were published in the last 25 years (1998 – 2023), with 101 (76%) published within the last 10 years (Fig. 2A).

**Table 1:** Study details and EEG-BCI paradigm design for all included studies. *CA*=Classification Accuracy, *CSP=*Common Spatial Patterns, *EEG=*Electroencephalography, *ERD=*Event-Related Desynchronisation, *ERSP*=Event-Related Spectral Perturbations, *FB*=Filter Bank*, FES=*Functional Electrical Stimulation*,* fMRI=Functional Magnetic Resonance Imaging, *LDA=*Linear Discriminant Analysis, *LL*=Lower Limb*, M*=Multimodal*, MI*=Motor Imagery, *U*=Unimodal*, UL*=Upper Limb, *SMC=*Sensorimotor Cortex, *SMR=*Sensorimotor Rhythm, *SVM=*Support Vector Machine*.*

**Study Details**		**Sensorimotor Task**		**Feedback Mode**		**EEG Neurofeedback Parameters**				**Offline EEG Analysis**			**BCI Performance**	
Title	Author and Year Published	MI / Motor Attempt / Other	UL / LL / UL and/or LL	Uni- / Multi-modal	Type(s)	Frequencies (Hz)	EEG channels	Signal Processing	EEG Feature	Frequencies (Hz)	EEG channels	EEG Feature	User performance measure(s)	Correlation with clinical motor outcome
Implicit Learning of a Finger Motor Sequence by Patients with Cerebral Palsy after Neurofeedback	Alves-Pinto et al., 2017	Other	−	U	Visual	Participant-specific band within 5–13	C3 & C4	−	ERD	4–35	C3 & C4	ERD	ERD enhancement	−
A Clinical Study of Motor Imagery-Based Brain-Computer Interface for Upper Limb Robotic Rehabilitation.	Ang et al., 2009	MI	UL	M	Visual & robotic	0.05–40	27	(FB)CSP	ERD	−	−	−	−	−
Clinical Study of Neurorehabilitation in Stroke Using EEG-Based Motor Imagery Brain-Computer Interface with Robotic Feedback	Ang et al., 2010	MI	UL	M	Visual & robotic	Not reported	27	(FB)CSP	Not reported	−	−	−	BCI CA	−
A Large Clinical Study on the Ability of Stroke Patients to Use an EEG-Based Motor Imagery Brain-Computer Interface	Ang et al., 2011	MI	UL	M	Visual & robotic	4–40 (4 Hz bins)	27	(FB)CSP Bayesian classifier	Not reported	−	−	−	BCI CA	−
A Randomized Controlled Trial of EEG-Based Motor Imagery Brain-Computer Interface Robotic Rehabilitation for Stroke	Ang et al., 2014a	MI	UL	M	Visual & robotic	4–40 (4 Hz bins)	27	(FB)CSP Bayesian classifier	Not reported	4–40	27	Brain symmetry index	−	−
Brain-Computer Interface-Based Robotic End Effector System for Wrist and Hand Rehabilitation: Results of a Three-Armed Randomized Controlled Trial for Chronic Stroke	Ang et al., 2014b	MI	UL	M	Visual & robotic	0.05–40	27	(FB)CSP	ERD	4–40	27	ERD	−	−
A Motor Imagery-Based Brain-Computer Interface Scheme for a Spinal Muscular Atrophy Subject in Cybathlon Race	Bao et al., 2021	MI	UL	U	Visual	4–40 (4 Hz bins)	16	(FB)CSP SVM	ERD	−	−	−	BCI CA	−
Plasticity of Premotor Cortico-Muscular Coherence in Severely Impaired Stroke Patients with Hand Paralysis	Belardinelli et al., 2017	MI	UL	U	Robotic	17–23 (Beta)	3 FC4, C4, CP4	−	ERD	18–30	32	Cortico-muscular coherence	−	−
Brain-Actuated Functional Electrical Stimulation Elicits Lasting Arm Motor Recovery after Stroke	Biasiucci et al., 2018	Motor attempt	UL	U	FES	4–40	16 (over SMC)	Gaussian classifier Laplacian	ERD	10–12 (Mu) 18–24 (Beta)	16 (over SMC)	Unspecified power spectral density features	BCI CA	Significant correlation with BCI CA
Rehabilitation of Patients with Cerebral Palsy Using Hand Exoskeleton Controlled by Brain Computer Interface	Bobrov et al., 2020	MI	UL	M	Visual & robotic	5–30	32	Bayesian classifier	ERD	5–30	32	ERD	BCI CA	−
Motor Imagery Impairment in Post-Acute Stroke Patients	Braun et al., 2017	MI	UL	U	Visual	8–30	24	CSP LDA classifier	ERD	5–35	C3 & C4	ERD ERD-based lateralisation index	BCI CA	−
Combination of Brain–Computer Interface Training and Goal-Directed Physical Therapy in Chronic Stroke: A Case Report	Broetz et al., 2010	MI	UL	M	Visual & robotic	Not reported (Mu)	Not reported (over ipsilesional SMC)	BCI2000 software system applied to both EEG and magnetoencephalography data	ERD	−	−	−	Mu power modulation (offline analysis performed using magnetoencephalography data only)	−
Contralesional Brain–Computer Interface Control of a Powered Exoskeleton for Motor Recovery in Chronic Stroke Survivors	Bundy et al., 2017	MI	UL	U	Robotic	8–12 (Mu) 12–30 (Beta)	C3 or C4 (depending on lesion)	−	ERD	0–30	8 F3, F4, T7, C3, Cz, C4, T8, Pz	ERD	1. Difference in hand position between movement and rest trials 2. ERD enhancement	Significant correlation with hand position Non-significant correlation with ERD enhancement
Chronic Stroke Recovery after Combined BCI Training and Physiotherapy: A Case Report	Caria et al., 2010	MI	UL	U	Robotic	Not reported (Mu)	Not reported (over ipsilesional SMC)	−	ERD	−	−	−	Proportion of trials with successful ERD enhancement	−
Longitudinal Analysis of Stroke Patients’ Brain Rhythms During an Intervention with a Brain-Computer Interface	Carino-Escobar et al., 2019	MI	UL	U	Robotic	8–32 (4 Hz bins)	4 F3, C3, T3, P3 or F4, C4, T4, P4 (depending on lesion)	(FB)CSP LDA classifier Particle swarm optimisation	ERD	8–13 (Alpha) 14–32 (Beta)	11	ERD	ERD enhancement	Significant correlation with alpha ERD/S enhancement
A Wireless BCI-FES Based on Motor Intent for Lower Limb Rehabilitation	Carrere et al., 2020	MI	LL	U	FES	8–30 (Mu & Beta)	Cz	BCI2000 software system	ERD	−	−	−	−	−
Effects of Brain-Computer Interface with Functional Electrical Stimulation for Gait Rehabilitation in Multiple Sclerosis Patients: Preliminary Findings in Gait Speed and Event-Related Desynchronization Onset Latency	Carrere et al., 2021	MI & motor attempt	LL	U	FES	8–30 (3 Hz bins)	8 C3, C4, T7, T8, Pz, F3, F4, Cz	Laplacian Autoregressive model for spectral power BCI2000 software system	ERD	8–30	Cz	ERD	1. BCI CA 2. ERD onset latency	−
Effect of Immersive Virtual Mirror Visual Feedback on Mu Suppression and Coherence in Motor and Parietal Cortex in Stroke	Chang et al., 2023	Motor attempt	UL	U	Visual	8–13 (Mu)	4 C3, C4, P3, P4	−	ERD	8–13 (Mu)	C3-P3 & C4-P4	ERD Magnitude squared coherence	−	−
Longitudinal Electroencephalography Analysis in Subacute Stroke Patients During Intervention of Brain–Computer Interface with Exoskeleton Feedback	Chen et al., 2020	Motor attempt	UL	U	Robotic	8–30 (Mu & Beta)	31	CSP LDA classifier	ERD	8–30	7 FC1, FC2, C3, CZ, C4, CP1, CP2	ERD	1. BCI CA 2. ERD enhancement	Informal correlation with ERD enhancement
EEG-Controlled Functional Electrical Stimulation Rehabilitation for Chronic Stroke: System Design and Clinical Application	Chen et al., 2021	MI	UL	M	Visual & FES	8–13 (Mu) 14–28 (Beta)	C3	CSP SVM	Not reported	8–30	32	ERD ERSP	1. ERD enhancement 2. Laterality coefficient values based on ERD	Significant correlation with laterality coefficient values based on mu ERD
Brain-Computer Interface-Based Soft Robotic Glove Rehabilitation for Stroke	Cheng et al., 2020	MI	UL	M	Visual & robotic	4–40 (4 Hz bins)	24	(FB)CSP Fisher's linear discriminant classifier	ERD	−	−	−	−	−
The Effect of Neurofeedback on a Brain Wave and Visual Perception in Stroke: A Randomized Control Trial	Cho et al., 2015	Other	−	M	Visual & auditory	12–18 (Beta) Reward feedback 0.5–4 (Delta) 22–36 (Beta) Inhibitory feedback	C5 or C6 (depending on lesion)	−	SMR power	4–50	Not reported	Beta power	Beta power modulation	−
Paired Associative Stimulation Using Brain-Computer Interfaces for Stroke Rehabilitation: A Pilot Study	Cho et al., 2016	MI	UL	M	Visual & FES	Not reported	45	CSP LDA classifier	ERD	8–12	C4 region	ERD	1. BCI CA 2. ERD enhancement	−
Functional Electrical Stimulation Controlled by Motor Imagery Brain-Computer Interface for Rehabilitation	Choi et al., 2020	MI	UL	U	FES	1–29 (4 Hz bins)	32	CSP LDA classifier SVM BCI2000 software system	ERD	−	−	−	1. BCI CA 2. Completion rate (how quickly MI task performed)	−
Active Physical Practice Followed by Mental Practice Using BCI-Driven Hand Exoskeleton: A Pilot Trial for Clinical Effectiveness and Usability	Chowdhury et al., 2018	Motor attempt	UL	M	Visual & robotic	8–12 (Mu) 16–24 (Beta)	C4 & CP4	CSP	ERD	8–24	12	ERD	1. BCI CA 2. ERD enhancement	Significant correlation with BCI CA
Corticomuscular Co-Activation Based Hybrid Brain-Computer Interface for Motor Recovery Monitoring	Chowdhury et al., 2020	Motor attempt	UL	M	Visual & robotic	8–12 (Mu) 15–30 (Beta)	12	SVM	ERD	8–30	12	ERD Cortico-muscular coherence	1. BCI CA 2. ERD enhancement	Significant correlation with mu/beta ERD values
Non-Invasive Brain–Computer Interface System: Towards its Application as Assistive Technology	Cincotti et al., 2008	MI or motor attempt	UL and/or LL	U	Visual	3–14	Subset of 59	BCI2000 software system	ERD	12–29	96	ERD Power spectral density using maximum entropy	BCI CA	−
An EEG-Based BCI Platform to Improve Arm Reaching Ability of Chronic Stroke Patients by Means of an Operant Learning Training with a Contingent Force Feedback	Cisotto et al., 2014	Motor attempt	UL	M	Visual, robotic & auditory	10–20 (Mu)	16 F3, Fz, F4, FC5, FC1, FC2, FC6, C3, Cz, C4, CP5, CP1, CP2, CP6, P3, P4	BCI2000 software system	ERD	6–20	16 F3, Fz, F4, FC5, FC1, FC2, FC6, C3, Cz, C4, CP5, CP1, CP2, CP6, P3, P4	ERD Power spectral density using maximum entropy	ERD enhancement	−
A Single Case Feasibility Study of Sensorimotor Feedback in Parkinson’s Disease	Cook et al., 2021	Other	−	U	Visual	12–17	26	−	SMR power	2–90	26	SMR power Beta power Burst rate Burst duration Interburst interval	1. SMR modulation 2. Beta power modulation	−
Residual Upper Arm Motor Function Primes Innervation of Paretic Forearm Muscles in Chronic Stroke after Brain-Machine Interface (BMI) Training	Curado et al., 2015	Motor attempt	UL	U	Robotic	8–13	16	−	ERD	−	−	−	−	−
Feasibility of a New Application of Non-Invasive Brain Computer Interface (BCI): A Case Study of Training for Recovery of Volitional Motor Control after Stroke	Daly et al., 2009	MI or motor attempt	UL	M	Visual & FES	5–30 (3 Hz bins)	58	BCI2000 software system	ERD	5–30	58	ERD Power spectral density using maximum entropy	BCI CA	−
On the Control of Brain-Computer Interfaces by Users with Cerebral Palsy	Daly et al., 2013	MI	UL and/or LL	U	Visual	9–29 (4 Hz bins)	16	LDA classifier	ERD	0–40	16	ERD	BCI CA	−
Investigating the Impact of Feedback Update Interval on the Efficacy of Restorative Brain–Computer Interfaces	Darvishi et al., 2017	MI	UL	U	Visual or robotic	15	5 FC4, CPz, CP4, PO4, TP8	Autoregressive model BCI2000 software system	ERD	−	−	−	−	−
Effects of Gamification in BCI Functional Rehabilitation	de Castro-Cros et al., 2020	MI	UL	M	Visual & FES	8–30	16	(FB)CSP LDA classifier	Not reported	−	−	−	BCI CA	−
Brain-Computer Interface Controlled Functional Electrical Stimulation Device for Foot Drop Due to Stroke	Do et al., 2012	Motor attempt	LL	U	FES	0.01–50 (2 Hz bins)	64	Bayesian classifier Approximate Information Discriminant Analysis	ERD	0.01–50	64	ERD	BCI CA	−
Evaluation of Neurofeedback Training in the Treatment of Parkinson's Disease: A Pilot Study	Erickson-Davis et al., 2012	Other	−	U	Auditory	8–15 (Alpha) Reward feedback 4–8 (Theta) 23–34 (Beta) Inhibitory feedback	C3 & C4	−	SMR power	1–30	32	Absolute/relative/cross-spectral power Peak frequency Amplitude asymmetry Phase-resets per second Coherence Phase lag Phase shift duration Burst rate Burst duration Interburst interval	SMR modulation	−
Assessment of the Efficacy of EEG-Based MI BCI With Visual Feedback and EEG Correlates of Mental Fatigue for Upper-Limb Stroke Rehabilitation	Foong et al., 2020	MI	UL	U	Visual	4–40	24	(FB)CSP Fisher's linear discriminant classifier	ERD	12–30 (Beta)	10 F3, Fz, F4, C3, Cz, C4, P3, Pz, P4, Oz	ERD	−	−
A New Gaze-BCI-Driven Control of an Upper Limb Exoskeleton for Rehabilitation in Real-World Tasks	Frisoli et al., 2012	MI	UL	U	Visual OR robotic	8–12 (Mu) 12–24 (Beta)	13 FC3, FCz, FC4, C5, C3, Cz, C2, C4, C6, CP3, CPz, CP4	CSP SVM	ERD	8–12 (Mu) 12–24 (Beta)	13 FC3, FCz, FC4, C5, C3, Cz, C2, C4, C6, CP3, CPz, CP4	ERD	BCI CA	−
Preliminary Results of a Controlled Study of BCI–Exoskeleton Technology Efficacy in Patients with Poststroke Arm Paresis	Frolov et al., 2016	MI	UL	M	Visual & robotic	5–30	32	Bayesian classifier	ERD	−	−	−	−	−
Post-Stroke Rehabilitation Training with a Motor-Imagery Based BCI-Controlled Hand Exoskeleton: A Randomised Controlled Multicentre Trial	Frolov et al., 2017	MI	UL	M	Visual & robotic	5–30	30	Bayesian classifier	Not reported	−	−	−	BCI CA	Significant correlation with BCI CA
Correlation Between the ERD in Grasp/Open Tasks of BCIs and Hand Function of Stroke Patients: A Cross Sectional Study	Fu et al., 2023	Motor attempt	UL	U	Robotic	8–30	10 FC3, CP3, C1, C3, C5, FC4, CP4, C2, C4, C6	CSP LDA classifier	ERD	8–40	C3 & C4	ERD ERSP	BCI CA	−
Closing the Sensorimotor Loop: Haptic Feedback Facilitates Decoding of Motor Imagery	Gomez-Rodriguez et al., 2011	MI	UL	M	Visual & robotic	2–42 (2 Hz bins)	35	Laplacian SVM BCI2000 software system	ERD	8–16 (Mu) 18–28 (Beta)	35	ERD	−	−
Treatment Effectiveness of Brain-Computer Interface Training for Patients with Focal Hand Dystonia: A Double-Case Study	Hashimoto et al., 2013	Motor attempt	UL	U	Visual	5–50 (2 Hz bins)	8 Surrounding C3 & C4	−	ERD	5–50	C3 & C4	ERD Cortico-muscular coherence	ERD enhancement	−
Functional Recovery from Chronic Writer’s Cramp by Brain-Computer Interface Rehabilitation: A Case Report	Hashimoto et al., 2014	Motor attempt	UL	U	Visual	5–50 (2 Hz bins)	8 Surrounding C3 & C4	LDA classifier Laplacian	ERD	5–50	C3 & C4	ERD Cortico-muscular coherence	ERD enhancement	−
Motor Imagery for Severely Motor-Impaired Patients: Evidence for Brain-Computer Interfacing as Superior Control Solution	Hohne et al., 2014	MI or motor attempt	UL and/or LL	U	Visual	0–45 (Mu & Beta) 0.2–4 (Lateralised readiness potential)	16 (over SMC)	CSP LDA classifier	ERD Lateralised readiness potential	8–40	16 (over SMC)	ERD Lateralised readiness potential	1. BCI CA 2. ERD & Lateralised readiness potential modulation	−
Using a Brain-Machine Interface to Control a Hybrid Upper Limb Exoskeleton During Rehabilitation of Patients with Neurological Conditions	Hortal et al., 2015	MI & motor attempt	UL	M	Robotic & FES	8–36 Hz (1 Hz bins; Mu & Beta)	16	Laplacian SVM	ERD	−	−	−	BCI CA	−
Motor Imagery-Based Brain-Computer Interface Combined with Multimodal Feedback to Promote Upper Limb Motor Function after Stroke: A Preliminary Study	Hu et al., 2021	MI	UL	M	Visual & sensory (brush)	8–30	11 FC3, FC4, C5, C3, C1, CZ, C2, C4, C6, CP3, CP4	−	ERD	5–30	11 FC3, FC4, C5, C3, C1, CZ, C2, C4, C6, CP3, CP4	ERD ERSP	ERD enhancement	−
Low Latency Estimation of Motor Intentions to Assist Reaching Movements Along Multiple Sessions in Chronic Stroke Patients: A Feasibility Study	Ibáñez et al., 2017	Motor attempt	UL	U	FES	7–30 (1 Hz bins) Bereitschaftspotential: 0.05–1	31	Bayes classifier Logistic regression classifier to combine outputs from ERD- and Bereitschaftspotential-based detectors	ERD Bereitschaftspotential	6–35 Bereitschaftspotential: 0.01–1	31	ERD	BCI CA	−
recoveriX: A New BCI-based Technology for Persons with Stroke	Irimia et al., 2016	MI	UL	M	Visual & FES	0.5–30	64	CSP LDA classifier	Not reported	Not reported	64	Unspecified power spectral density features	−	−
Brain-Computer Interfaces with Multi-Sensory Feedback for Stroke Rehabilitation: A Case Study	Irimia et al., 2017	MI	UL	M	Visual & FES	8–30	45	CSP LDA classifier	ERD	8–12	45	ERD	1. BCI CA 2. ERD enhancement	−
High Classification Accuracy of a Motor Imagery Based Brain-Computer Interface for Stroke Rehabilitation Training	Irimia et al., 2018	MI	UL	M	Visual & FES	0.5–30	64	CSP LDA classifier	Not reported	−	−	−	BCI CA	−
BCI-Activated Electrical Stimulation in Children with Perinatal Stroke and Hemiparesis: A Pilot Study	Jadavji et al., 2023	MI	UL	M	Visual & FES	0.5–20 (Mu, Alpha & Beta)	16	CSP LDA classifier	ERD	−	−	−	BCI CA (& Cohen's Kappa)	−
Tailoring Brain–Machine Interface Rehabilitation Training Based on Neural Reorganization: Towards Personalized Treatment for Stroke Patients	Jia et al., 2022	Motor attempt	UL	U	Robotic	0.5–40	31	Unspecified classifier	ERD	0.5–40	31	ERD ERD-based lateralisation index	1. ERD enhancement 2. Lateralisation index	−
Restoration of Upper Limb Function after Chronic Severe Hemiplegia: A Case Report on the Feasibility of a Brain-Computer Interface-Triggered Functional Electrical Stimulation Therapy	Jovanovic et al., 2020	Motor attempt	UL	U	FES	8–12 (Mu)	C2	−	ERD	−	−	−	−	−
Initial Experience with a Sensorimotor Rhythm-Based Brain-Computer Interface in a Parkinson’s Disease Patient	Kasahara et al., 2018	MI	UL	U	Visual	9.5–12.5 (Mu)	C3 & C4	Autoregressive model BCI2000 software system	ERD	1–25	C3 & C4	ERD	Success rate (% of successful trials)	−
Oscillatory Neurofeedback Networks and Post-Stroke Rehabilitative Potential in Severely Impaired Stroke Patients	Kern et al., 2023	MI	UL	U	Robotic	16–22 (2 Hz bins; Beta)	3 FC4, C4, CP4	Linear classifier Autoregressive model for spectral power BCI2000 software system	ERD	14–24 (ERSP) 6–16 (phase slope index)	24	ERD ERSP Phase slope index	−	−
Rewiring Cortico-Muscular Control in the Healthy and Post-Stroke Human Brain with Proprioceptive B-Band Neurofeedback	Khademi et al., 2022	MI	UL	U	Robotic	16–22 (Beta)	3 CP4, C4, FC4	Linear classifier Autoregressive model for spectral power BCI2000 software system	ERD	2–46 (1 Hz bins)	32	ERD Cortico-muscular coherence	1. ERD enhancement 2. CMC modulation	Significant correlation with CMC modulation
Patients with ALS Can Use Sensorimotor Rhythms to Operate a Brain-Computer Interface	Kübler et al., 2005	MI	UL and/or LL	U	Visual	8–12 (Mu) or 18–26 (Beta)	1 CP3, CP4 or Cz	Autoregressive model	ERD	−	−	−	BCI CA	−
Short Term Priming Effect of Brain-Actuated Muscle Stimulation Using Bimanual Movements in Stroke	Kumari et al., 2022	Motor attempt	UL	U	Visual	Participant-specific band within 8–30 (4 Hz bins)	1 or 2 bipolar FC3−CP3 and/or FC4−CP4 (depending on lesion and session type)	−	ERD	2–40	1 or 2 bipolar FC3−CP3 and/or FC4 −CP4 (depending on lesion and session type)	ERD ERSP ERD-based lateralisation index Brain symmetry index Delta alpha ratio	Lateralisation index	−
Neurofeedback Training Improves the Dual-Task Performance Ability in Stroke Patients	Lee et al., 2015	Other	−	U	Visual	12–15 (Beta) Reward feedback 1–4 (Delta) 43–50 (Gamma) Inhibitory feedback	Cz	−	SMR power	−	−	−	SMR modulation	−
Transferring Brain–Computer Interfaces Beyond the Laboratory: Successful Application Control for Motor-Disabled Users	Leeb et al., 2013	MI	UL and/or LL	U	Visual	7–13 (Mu) 13–30 (Beta)	1–6 from 16 Fz, FC3, FC1, FCz, FC2, FC4, C3, C1, Cz, C2, C4, CP3, CP1, CPz, CP2, CP4	Gaussian classifier Laplacian	ERD	7–30	3 C3, C4, Cz	ERD	−	−
Neurophysiological Substrates of Stroke Patients with Motor Imagery-Based Brain-Computer Interface Training	Li et al., 2013	MI	UL	M	Visual, FES & auditory	8–30	16 (over SMC)	CSP SVM	ERD	8–30	16 (over SMC)	ERD	1. BCI CA 2. ERD enhancement	Significant correlation with strength of ERD
Sensorimotor Rhythm-Brain Computer Interface with Audio-Cue, Motor Observation and Multisensory Feedback for Upper-Limb Stroke Rehabilitation: A Controlled Study	Li et al., 2022	MI	UL	M	Visual, robotic & auditory	8–12 (Mu)	C3 or C4	−	ERD	8–12	C3 & C4	ERD	ERD enhancement	Non-significant correlation with strength of mu ERD
A Multi-Target Motor Imagery Training Using Bimodal EEG-FMRI Neurofeedback: A Pilot Study in Chronic Stroke Patients	Lioi et al., 2020	MI	UL	U	Visual	8–30	18 (bimodal EEG & fMRI) 8 (unimodal EEG)	CSP or Laplacian	ERD	8–30	18 (bimodal EEG & fMRI) 5 (unimodal EEG)	ERD	ERD enhancement	Informal correlation with ERD enhancement
The Impact of Neurofeedback on Effective Connectivity Networks in Chronic Stroke Patients: An Exploratory Study	Lioi et al., 2021	MI	UL	U	Visual	8–30	18 (bimodal EEG & fMRI) 8 (unimodal EEG)	CSP Laplacian	ERD	−	−	−	−	−
Motor Imagery Based Brain-Computer Interface Control of Continuous Passive Motion for Wrist Extension Recovery in Chronic Stroke Patients	Lu et al., 2020	MI	UL	M	Visual & robotic	8–13 (Alpha) 14–30 (Beta)	24	−	ERD	8–30	24	ERD	1. BCI classification rate (unspecified) 2. ERD enhancement	−
BCI-Triggered Functional Electrical Stimulation Therapy for Upper Limb	Marquez-Chin et al., 2016a	Motor attempt	UL	U	FES	18–28 (Beta)	Fz	−	ERD	−	−	−	−	−
EEG-Triggered Functional Electrical Stimulation Therapy for Restoring Upper Limb Function in Chronic Stroke with Severe Hemiplegia	Marquez-Chin et al., 2016b	Motor attempt	UL	U	FES	18–28 (Beta)	Fz	−	ERD	4–30	6 F3, Fz, F4, C3, Cz, C4	ERD	Success rate (of UL movement)	−
Real-Time Control of a Video Game with a Direct Brain–Computer Interface	Mason et al., 2004	Other	−	U	Visual	0.1–30	6 bipolar F1-FC1, Fz-FCz, F2-FC2, FC1-C1, FCz-Cz, FC2-C2	Low frequency asynchronous switch design classifier	Not reported	−	−	−	BCI CA	−
Brain-Controlled Functional Electrical Stimulation for Lower-Limb Motor Recovery in Stroke Survivors	McCrimmon et al., 2014	Motor attempt	LL	U	FES	8–30	32	LDA or Approximate Information Discriminant Analysis Bayesian classifier	Not reported	8–30	32	Unspecified power spectral density features	−	−
Brain-Controlled Functional Electrical Stimulation Therapy for Gait Rehabilitation after Stroke: A Safety Study	McCrimmon et al., 2015	Motor attempt	LL	U	FES	8–30 (2 Hz bins)	1 Cz, C5, or CPz	−	ERD	8–30	32	ERD	ERD enhancement	Informal correlation with ERD enhancement
Emulation Of Computer Mouse Control with a Non-Invasive Brain-Computer Interface	McFarland et al., 2008	MI	UL	U	Visual	8–30 (3 Hz bins)	2 C3, C4, CP3, CP4 or FC1	Laplacian Autoregressive model for spectral power BCI2000 software system	ERD	0.1–60	64	ERD	Success rate (% target attainment)	−
BCI-Based Rehabilitation on the Stroke in Sequela Stage	Miao et al., 2020	MI	UL	M	Visual & FES	8–30	16	CSP LDA classifier	ERD	8–30	C3 & C4	ERD	1. BCI CA 2. ERD enhancement	−
Answering Questions with an Electroencephalogram-Based Brain-Computer Interface	Miner et al., 1998	Other	−	M	Visual & auditory	8–12 (Mu) or 18–25 (Beta)	C3 & C4	Laplacian Autoregressive model	ERD	8–25	64	Unspecified power spectral density features	Success rate (% target attainment)	−
Brain–Computer Interface: The First Experience of Clinical Use in Russia	Mokienko et al., 2016	MI	UL	M	Visual & robotic	5–30 (Alpha or Beta)	30	Bayes classifier	ERD	5–30	30	ERD	BCI CA (& Cohen's Kappa)	−
Neurofeedback Training of Alpha-Band Coherence Enhances Motor Performance	Mottaz et al., 2015	Other	−	U	Visual	8–12 (Alpha)	Not reported (over ipsilesional SMC)	−	Functional connectivity (alpha)	1–20	128	Imaginary component of coherence	Functional connectivity (alpha) change	Significant correlation with functional connectivity change
Modulating Functional Connectivity after Stroke with Neurofeedback: Effect on Motor Deficits in a Controlled Cross-Over Study	Mottaz et al., 2018	Other	−	U	Visual	8–12 (Alpha)	Not reported (over ipsilesional SMC)	−	Functional connectivity (alpha)	1–20	128	Imaginary component of coherence	Functional connectivity (alpha) change	Significant correlation with functional connectivity change
Efficacy of Brain-Computer Interface-Driven Neuromuscular Electrical Stimulation for Chronic Paresis after Stroke	Mukaino et al., 2014	Motor attempt	UL	U	NMES	Not reported (Mu & Beta)	5 Surrounding C3 & C4	−	ERD	0.5–60	5 Surrounding C3 & C4	ERD Cortico-muscular coherence	ERD enhancement	Informal correlation with ERD
EEG-Based Neuroprosthesis Control: A Step Towards Clinical Practice	Muller-Putz et al., 2005	MI	UL and/or LL	M	Visual & robotic	12–14 (Beta) 18–22 (Beta)	2 bipolar Surrounding Cz & C4	LDA classifier	ERD	0.5–32	2 bipolar Cz & C4	ERD	BCI CA	−
Effect of Auditory Neurofeedback Training on Upper Extremity Function and Motor Imagery Ability in a Stroke Patient: A Single Case Study	Nakano et al., 2018	MI	UL	U	Auditory	8–13 (Mu)	C3 & C4	−	ERD	−	−	−	−	−
Restoring Activities of Daily Living Using an EEG/EOG-Controlled Semiautonomous and Mobile Whole-Arm Exoskeleton in Chronic Stroke	Nann et al., 2021	Motor attempt	UL	U	Robotic	8–12 0.1–5 (EOG)	C3 or C4	Laplacian BCI2000 software system	ERD	1–30	5 F3, T3, C3, Cz, P3 or F4, T4, C4, Cz, P4 (depending on lesion)	ERD	ERD onset latency	−
Reinforcement Learning of Self-Regulated Beta-Oscillations for Motor Restoration in Chronic Stroke	Naros & Gharabaghi, 2015	MI	UL	U	Robotic	17–23 (2 Hz bins; Beta)	3 FC4, C4, CP4	LDA classifier Bayesian model for threshold adaptation Autoregressive model for spectral power BCI2000 software system	ERD	3–120	32	ERD ERSP	ERD enhancement	Informal correlation with ERD
Clinical Application of an EEG-Based Brain–Computer Interface: A Case Study in a Patient with Severe Motor Impairment	Neuper et al., 2003	MI	UL	M	Visual & auditory	20–30 (Beta)	1 bipolar Surrounding C3	LDA classifier	ERD	5–30 (Beta)	2 bipolar Surrounding C3	ERD	BCI CA	−
Feasibility of Task-Specific Brain-Machine Interface Training for Upper-Extremity Paralysis in Patients with Chronic Hemiparetic Stroke	Nishimoto et al., 2018	MI	UL	M	Visual, robotic & NMES	8–13 (Mu)	1 bipolar C3 or C4 (depending on lesion)	−	ERD	−	−	−	Number of ERD detections	Some significant correlations with number of ERD detections
Controlling Pre-Movement Sensorimotor Rhythm Can Improve Finger Extension after Stroke	Norman et al., 2018	MI & motor attempt	UL	M	Visual & robotic	12–24 (3 Hz bins)	1–3 bipolar C3−Cz, C4−Cz, CP3−Cz or CP4−Cz	Autoregressive model BCI2000 software system	ERD	12–24	16	ERD	Success rate (% of successful trials)	Non-significant correlation with success rate
Neurofeedback Training and Physical Training Differentially Impacted on Reaction Time and Balance Skills Among Iranian Veterans with Spinal Cord Injury	Norouzi & Vaezmousavi, 2019	MI	UL	U	Visual	12–15 (Beta)	C3 & C4	−	SMR power	−	−	−	−	−
Functional Recovery in Upper Limb Function in Stroke Survivors by Using Brain-Computer Interface a Single Case A-B-A-B Design	Ono et al., 2013	Motor attempt	UL	U	NMES	Not reported (Alpha & Beta)	10 Surrounding C3 and C4	LDA classifier	ERD	2–100	10 Surrounding C3 & C4	ERD Cortico-muscular coherence	ERD enhancement	−
Brain-Computer Interface with Somatosensory Feedback Improves Functional Recovery from Severe Hemiplegia Due to Chronic Stroke	Ono et al., 2014	Motor attempt	UL	U	Visual OR robotic	Participant-specific band or 9–12	1 bipolar Participant-specific or C3-C3a	−	ERD	Not reported	10	ERD	ERD enhancement	−
Multimodal Sensory Feedback Associated with Motor Attempts Alters Bold Responses to Paralyzed Hand Movement in Chronic Stroke Patients	Ono et al., 2015	Motor attempt	UL	U	Visual OR robotic	Participant-specific band or 9–12	1 bipolar Participant-specific or C3-C3a	−	ERD	2–60	10	ERD	ERD enhancement	−
Hand Motor Rehabilitation of Patients with Stroke Using Physiologically Congruent Neurofeedback	Ono et al., 2018	MI	UL	U	Robotic	8–13 (Mu)	C3 or C4 (depending on lesion)	−	ERD	0.5–30	C3 & C4	ERD	ERD enhancement	Non-significant correlation with ERD enhancement
Rehabilitation of Hand in Subacute Tetraplegic Patients Based on Brain Computer Interface and Functional Electrical Stimulation: A Randomised Pilot Study	Osuagwu et al., 2016	Motor attempt	UL	M	Visual & FES	7–30 (Mu & Beta)	3 bipolar CP3–CF3, CPz–CFz, CP4–CF4	LDA classifier	ERD	0.5–60	48	ERD	ERD enhancement	−
Brain Oscillations Control Hand Orthosis in a Tetraplegic	Pfurtscheller et al., 2000	MI	UL and/or LL	U	Visual OR robotic	0.5–30	2 bipolar Surrounding C4, C3, Cz	LDA classifier Autoregressive model for spectral power	SMR power	0.5–30	60	SMR power	BCI CA	−
Brain–Computer Interface Boosts Motor Imagery Practice During Stroke Recovery	Pichiorri et al., 2015	MI	UL	U	Visual	1–45	51	BCI2000 software system	ERD	1–45	51	ERD Partial directed coherence	1. Success rate (% of successful trials) 2. ERD enhancement 3. Partial directed coherence	Significant correlation with connectivity change
An All-In-One BCI-Supported Motor Imagery Training Station: Validation in A Real Clinical Setting with Chronic Stroke Patients	Pichiorri et al., 2019	MI	UL	U	Visual	Participant-specific band within 1–45	31 (over SMC) Participant-specific	−	ERD	1–45	31 (over SMC)	ERD	ERD enhancement	−
Evaluating Person-Centered Factors Associated with Brain–Computer Interface Access to a Commercial Augmentative and Alternative Communication Paradigm	Pitt & Brumberg, 2022	MI or motor attempt	UL and/or LL	U	Visual	Participant-specific band within 8–25	62	CSP LDA classifier	SMR power	−	−	−	BCI CA (& Cohen's Kappa)	−
Applying a Brain-Computer Interface to Support Motor Imagery Practice in People with Stroke for Upper Limb Recovery: A Feasibility Study	Prasad et al., 2010	MI	UL	U	Visual	Participant-specific bands within 8–12 (Mu) & 18–25 (Beta)	2 bipolar Surrounding C3 & C4	Fuzzy logic system classifier Autoregressive model for spectral power	ERD	8–12 (Mu) 18–25 (Beta)	2 bipolar C3 & C4	ERD	1. BCI CA 2. ERD enhancement	Non-significant correlation with ERD/S ratios
Brain–Machine Interface in Chronic Stroke Rehabilitation: A Controlled Study	Ramos-Murguialday et al., 2013	Motor attempt	UL	U	Robotic	8–13 (Alpha)	Subset of 16 (over ipsilesional SMC)	−	SMR power	−	−	−	Success rate (of UL movement)	−
Brain-Machine Interface in Chronic Stroke: Randomized Trial Long-Term Follow-Up *(Follow-up study of the above)*	Ramos-Murguialday et al., 2019	Motor attempt	UL	U	Robotic	8–13 (Alpha)	Subset of 16 (over ipsilesional SMC)	−	SMR power	−	−	−	−	−
Effect of Neurofeedback and Electromyographic-Biofeedback Therapy on Improving Hand Function in Stroke Patients	Rayegani et al., 2014	MI	UL	M	Visual & auditory	12–18 (SMR) Reward feedback 4–8 (Theta) 13–30 (Beta) Inhibitory feedback	C3	−	SMR power	12–18	C3	SMR power	SMR modulation	−
Shutting Down Sensorimotor Interferences after Stroke: A Proof-Of-Principle SMR Neurofeedback Study	Reichert et al., 2016	Other	−	U	Visual	12–15	Cz	−	SMR power	12–15	5 Cz, CPz, Pz, Poz, FCz	SMR power Coherence Event-related potential	SMR & Event-related potential modulation	−
Behavioral Outcomes Following Brain–Computer Interface Intervention for Upper Extremity Rehabilitation in Stroke: A Randomized Controlled Trial	Remsik et al., 2018	Motor attempt	UL	M	Visual, FES & tongue stimulation	8–12 (Mu) 16–24 (Beta)	16 (over SMC)	BCI2000 software system	SMR power	−	−	−	−	−
Ipsilesional Mu Rhythm Desynchronization and Changes in Motor Behaviour Following Post Stroke BCI Intervention for Motor Rehabilitation	Remsik et al., 2019	Motor attempt	UL	M	Visual, FES & tongue stimulation	8–12 (Mu) 18–26 (Beta)	C3 & C4	BCI2000 software system	ERD	4–30 (Mu & Beta)	16	ERD R-Squared coherence ERD-based lateralisation index	1. ERD enhancement 2. Lateralisation index	Non-significant correlation with mu ERD enhancement
Ipsilesional Mu Rhythm Desynchronization Correlates with Improvements in Affected Hand Grip Strength and Functional Connectivity in Sensorimotor Cortices Following BCI-FES Intervention for Upper Extremity in Stroke Survivors	Remsik et al., 2021	Motor attempt	UL	M	Visual & FES	8–12 (Mu)	3 C3, C4, Cz	BCI2000 software system	ERD	4–30 (Mu & Beta)	16	ERD Isolated effective partial coherence	1. Success rate (% of successful trials) 2. ERD enhancement	Significant correlation with mu ERD enhancement
Combining a Hybrid Robotic System with a Bain-Machine Interface for the Rehabilitation of Reaching Movements: A Case Study with a Stroke Patient	Resquin et al., 2016	Motor attempt	UL	M	Visual, robotic & FES	7–30 Bereitschaftspotential: < 2	28 Bereitschaftspotential: average potential of Cz & C2 minus F3, Fz, F4, C3, C4, P3, Pz, P4	Laplacian Bayesian classifier Logistic regression classifier to combine outputs from ERD- and Bereitschaftspotential −based detectors	ERD Bereitschaftspotential	−	−	−	BCI CA	−
Applying Action Observation During a Brain-Computer Interface on Upper Limb Recovery in Chronic Stroke Patients	Rungsirisilp et al., 2023	MI	UL	U	FES	8–30	10 FC3, FC4, C5, C6, C3, C4, C1, C2, CP3, CP4 (depending on lesion)	CSP LDA classifier	ERD	8–13 (Alpha) 14–30 (Beta)	C3 or C4	ERD	1. BCI CA 2. ERD enhancement	Non-significant correlation with BCI CA Significant correlation with beta ERD strength Non-significant correlation with alpha ERD strength
Examination of Effectiveness of Kinaesthetic Haptic Feedback for Motor Imagery-Based Brain-Computer Interface Training	Sakamaki et al., 2022	MI	UL	U	Visual OR robotic	7–30	8 Cz, Cp, F3, C3, P3, F4, C4, P4	CSP LDA classifier	ERD	8–26	8	ERD	BCI CA	−
Brain Computer Interface Treatment for Motor Rehabilitation of Upper Extremity of Stroke Patients − A Feasibility Study	Sebastian-Romagosa et al., 2020	MI	UL	M	Visual & FES	8–30	16	CSP LDA classifier	Not reported	−	−	−	BCI CA	Significant correlation with BCI CA
Effects of Neurofeedback Training with an Electroencephalogram-Based Brain Computer Interface for Hand Paralysis in Patients with Chronic Stroke − A Preliminary Case Series Study	Shindo et al., 2011	MI	UL	M	Visual & robotic	8–16 (Alpha) 16–26 (Beta)	2 bipolar C3-C3A & C4-C4A	LDA classifier	ERD	8–16 (Alpha) 16–26 (Beta)	4 bipolar Surrounding C3 & C4	ERD	1. Success rate (% of successful trials) 2. ERD enhancement	−
Kinematic and Neurophysiological Consequences of an Assisted-Force-Feedback Brain-Machine Interface Training: A Case Study	Silvoni et al., 2013	Motor attempt	UL	M	Visual, robotic & auditory	Affected UL: 14–17 Unaffected UL: 11–14	Affected UL: C3, Cp1, P3, Cp5 Unaffected UL: C4, Cp2, P4, Cp6	BCI2000 software system	ERD	0.5–30 (3 Hz bins)	8 C3, CP1, P3, CP5, C4, CP2, P4, CP6	ERD	1. Success rate (% of successful trials) 2. ERD enhancement	−
Brain-Computer Interface Training with Functional Electrical Stimulation: Facilitating Changes in Interhemispheric Functional Connectivity and Motor Outcomes Post-Stroke	Sinha et al., 2021	Motor attempt	UL	M	Visual, FES & tongue stimulation	8–12 (Mu) 18–25 (Beta)	C3 & C4	Linear classifier Autoregressive model for spectral power BCI2000 software system	SMR power	−	−	−	−	−
Exploring Self-Paced Embodiable Neurofeedback for Post-Stroke Motor Rehabilitation	Spychala et al., 2020	MI	UL	U	Visual	8–28	24	CSP Logistic regression classifier	ERD	5–50	24	ERD	−	−
Neurological Rehabilitation of Stroke Patients via Motor Imaginary-Based Brain-Computer Interface Technology	Sun et al., 2011	MI	UL and/or LL	U	Visual	8–30	13	−	ERD	8–30	5 CP4, C4, Cz, C3, CP3	ERD	−	−
Neurorehabilitation Therapy of Patients with Severe Stroke Based on Functional Electrical Stimulation Commanded by a Brain Computer Interface	Tabernig et al., 2018	MI	UL	U	FES	0.2–43	8 F3, F4, T7, T8, C3, C4, Cz, Pz	BCI2000 software system	ERD	8–30	C3 & C4	ERD	−	−
Event Related Desynchronization-Modulated Functional Electrical Stimulation System for Stroke Rehabilitation: A Feasibility Study	Takahashi et al., 2012	Motor attempt	LL	M	Visual & FES	24–26 (Beta)	1 bipolar FCz – CPz	−	ERD	−	−	−	−	−
Post-Acute Stroke Patients Use Brain-Computer Interface to Activate Electrical Stimulation	Tan et al., 2010	MI or motor attempt	UL	M	Visual & NMES	8–12 (Mu)	6 FC3, FC4, C3, C4, CP3, CP4	−	ERD	8–25	6 FC3, FC4, C3, C4, CP3, CP4	ERD	−	−
Sensorimotor Connectivity after Motor Exercise with Neurofeedback in Post-Stroke Patients with Hemiplegia	Tsuchimoto et al., 2019	MI	UL	M	Robotic & NMES	Participant-specific band within 8–13 (Alpha) 15–23 (Beta)	1 bipolar Surrounding C3 or C4 (depending on lesion)	LDA classifier	ERD	8–13 (Alpha) 15–23 (Beta)	2 bipolar C3 & C4	ERD	−	−
Resting State Changes in Functional Connectivity Correlate with Movement Recovery for BCI And Robot-Assisted Upper-Extremity Training after Stroke	Varkuti et al., 2013	MI	UL	M	Visual & robotic	Not reported	27	(FB)CSP	Not reported	−	−	−	−	−
Efficacy and Brain Imaging Correlates of an Immersive Motor Imagery BCI-Driven VR System for Upper Limb Motor Rehabilitation: A Clinical Case Report	Vourvopoulos et al., 2019a	MI	UL	M	Visual, auditory & vibrotactile	8–12 (Mu) 12–30 (Beta)	C3 or C4	CSP LDA classifier Bayesian model for threshold adaptation	ERD	8–12 (Mu) 12–30 (Beta)	C3 & C4	ERD ERSP ERD-based lateralisation index	1. BCI CA 2. ERD enhancement 3. Lateralisation index	−
Effects of a Brain Computer Interface with Virtual Reality (VR) Neurofeedback: A Pilot Study in Chronic Stroke Patients	Vourvopoulos, et al., 2019b	Motor attempt	UL	U	Visual	8–12 (Mu) 12–30 (Beta)	C3 or C4	Laplacian	ERD	8–12 (Mu) 12–30 (Beta)	C3 & C4	ERD ERSP ERD-based lateralisation index	1. Success rate (% of successful trials) 2. ERD enhancement	−
Multimodal Head-Mounted Virtual-Reality Brain-Computer Interface for Stroke Rehabilitation	Vourvopoulos et al., 2019c	Motor attempt	UL	U	Visual	8–12 (Mu) 12–30 (Beta)	C3 or C4	−	ERD	8–12 (Mu) 12–30 (Beta)	C3 & C4	ERD ERSP	Success rate (% of successful trials)	−
Development of a Brain-Machine Interface for Stroke Rehabilitation Using Event-Related Desynchronization and Proprioceptive Feedback	Wada et al., 2019	MI	UL	M	Visual & robotic	8–13 (Mu)	C3 or C4	−	ERD	2–14	C3 & C4	ERD	ERD enhancement	−
Differentiated Effects of Robot Hand Training with and without Neural Guidance on Neuroplasticity Patterns in Chronic Stroke	Wang et al., 2018	MI	UL	U	Robotic	8–13 (Mu)	C3 or C4 (depending on lesion)	LDA classifier	ERD	2–48	16	ERD	BCI CA	Non-significant correlation with BCI CA
Multimodal Neural Response and Effect Assessment During a BCI-Based Neurofeedback Training after Stroke	Wang et al., 2022	MI	UL	M	Visual, FES & auditory	8–13 (Alpha) 14–29 (Beta)	C3 & C4	CSP SVM	ERD	5–35	64	ERD ERSP	ERD enhancement	−
Control of a Two-Dimensional Movement Signal by a Non-Invasive Brain-Computer Interface in Humans	Wolpaw & McFarland, 2004	Other	−	U	Visual	8–12 (Mu) 18–26 (Beta)	C3 & C4	Laplacian Autoregressive model for spectral power	SMR power	8–12 (Mu) 18–26 (Beta)	C3 & C4	SMR power	−	−
Brain Functional Networks Study of Subacute Stroke Patients with Upper Limb Dysfunction after Comprehensive Rehabilitation Including BCI Training	Wu et al., 2020	MI	UL	M	Visual & robotic	8–13 (Mu)	C3 & C4	−	ERD	−	−	−	−	−
Case Report: Post-Stroke Interventional BCI Rehabilitation in an Individual with Pre-existing Sensorineural Disability	Young et al., 2014	Motor attempt	UL	M	Visual, FES & tongue stimulation	Not reported (Mu & Beta)	5 C3, CP3, Cz, C4, CP4	BCI2000 software system	ERD	−	−	−	Success rate (% of target attainment)	−
BCI Training Effects on Chronic Stroke Correlate with Functional Reorganization in Motor-Related Regions: A Concurrent EEG And FMRI Study	Yuan et al., 2021	MI	UL	M	Visual & robotic	8–13 (Alpha)	C3 or C4 (depending on lesion)	−	ERD	8–12 (Alpha) 12–30 (Beta)	64	ERD Generalised partial directed coherence	−	−
Brain-Computer Interface Combined with Mental Practice and Occupational Therapy Enhances Upper Limb Motor Recovery, Activities of Daily Living, and Participation in Subacute Stroke	Zanona et al., 2023	MI	UL	M	Visual & robotic	8–13 (Mu)	6 FC3, C3, CP3, FC4, C4, CP4	−	ERD	8–13 (Mu)	14 FC3, FC1, FCz, FC2, FC4, C1, C3, CP1, CP3, FC4, C2, C4, CP2, CP4, Cz	ERD	−	−
EEG-Based Brain Network Analysis of Chronic Stroke Patients after BCI Rehabilitation Training	Zhan et al., 2022	MI	UL	M	Visual, FES & auditory	8–13 (Alpha)	22	−	Not reported	8–13 (Alpha)	22	Directed transfer function Global/local efficiency Clustering coefficient Node strength Network density	−	−
Combining Mental Training and Physical Training with Goal-Oriented Protocols in Stroke Rehabilitation: A Feasibility Case Study	Zhang et al., 2018	MI & motor attempt	UL	M	Robotic & FES	1–45	32	(FB)CSP LDA classifier Dual Augmented Lagrangian SVM BCI2000 software system	ERD	6–35 (Mu & Beta)	32	ERD Brain symmetry index	Success rate (% of successful trials)	−
An Adaptive Brain-Computer Interface to Enhance Motor Recovery after Stroke	Zhang et al., 2023	MI	UL	M	Visual & FES	8–30	30	CSP SVM	Not reported	8–12 (Alpha) 13–30 (Beta)	30	Directed transfer function Global efficiency Clustering coefficient	1. BCI CA 2. Failure rate (% of unsuccessful trials)	Informal correlation with BCI CA
High-Intensity Chronic Stroke Motor Imagery Neurofeedback Training at Home: Three Case Reports	Zich et al., 2017	MI	UL	U	Visual	8–30	24	CSP LDA classifier	ERD	8–30	8 C3, CP1, CP5, P3, C4, CP2, CP6, P4	ERD ERD-based lateralisation index	1. ERD enhancement 2. Lateralisation index	−
On The Way Home: A BCI‐FES Hand Therapy Self‐Managed by Sub‐Acute SCI Participants and Their Caregivers: A Usability Study	Zulauf-Czaja et al., 2021	Motor attempt	UL	M	Visual & FES	8–12 (Alpha)	2 bipolar FC3 and CP3 or FC4 and CP4	−	ERD	8–24	16	ERD ERSP	BCI CA	−

### 3.2 Study design

The study design of included articles was categorised based on the Oxford Centre for Evidence-Based Medicine (OCEBM) (OCEBM, 2011). Most studies were case-series (n = 57, 43%) or case reports of three or less patients (n = 39, 29%), which we categorised as level 4. Of these level 4 studies, 32 reported single patient case reports and 43 studies enrolled between two and ten patients in an intervention condition. Although a proportion of articles reported randomised controlled trials (n = 37, 28%), these were mostly unpowered studies without sample size estimations. Four articles reported enrolling sample sizes large enough to achieve a statistical power of 80 – 95% (Frolov et al., 2017, Norouzi and Vaezmousavi, 2019, Tsuchimoto et al., 2019, Zanona et al., 2023). However, the derivation of these power analyses was sometimes unclear.

All three studies enrolling children were level 4 case series (Cincotti et al., 2008, Bobrov et al., 2020, Jadavji et al., 2023).

### 3.3 Participant age and motor impairment

The neurological motor impairment diagnoses of participants enrolled in each study are shown in Fig. 2B. Adult-onset stroke was most common (n = 103, 77%), followed by CP (n = 6, 5%) (Alves-Pinto et al., 2017, Bobrov et al., 2020, Daly et al., 2013, Jadavji et al., 2023, Neuper et al., 2003, Sakamaki et al., 2022) and spinal cord injury (n = 6, 5%) (Mason et al., 2004, McFarland et al., 2008, Muller-Putz et al., 2005, Norouzi and Vaezmousavi, 2019, Wolpaw and McFarland, 2004, Zulauf-Czaja et al., 2021).

Most studies enrolled adult participants only (n = 130, 98%). Three studies included children with neurological motor impairments, one of which enrolled individuals aged 12 – 55 years (mean 29.3 years) with either Spinal Muscular Atrophy II (n = 8, including 2 children) or Duchenne Muscular Dystrophy (n = 6, including 2 children) and 14 healthy controls (Cincotti et al., 2008). Only two studies focused solely on children: one enrolled 14 children with CP (10 hemiplegic, 3 spastic diplegic and 1 tetraplegic; mean age 13.7 years) (Bobrov et al., 2020); the other enrolled 13 children with hemiparetic CP (mean age 12.2 years) (Jadavji et al., 2023).

### 3.4 Sensorimotor task parameters

**Fig. 3:**
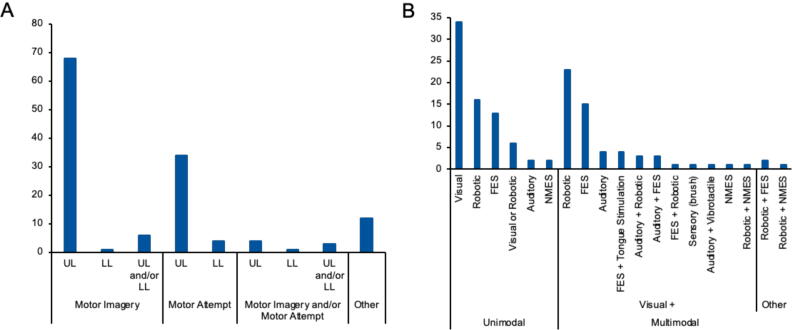
(A) Sensorimotor task parameters. LL=Lower Limb, UL=Upper Limb. (B) Neurofeedback mode(s) employed in studies. As noted in the text, haptic feedback was delivered via multiple methods including robotic devices, vibrotactile and brush stimuli.

The sensorimotor task parameters employed in each study are displayed in Fig. 3A, including limb(s) involved and sensorimotor task. Most commonly, participants were asked to perform motor imagery (MI) only (n = 75, 56%). Other studies involved participants attempting or executing actual movement (n = 38, 29%). A portion of studies explored both paradigms (n = 9, 7%). In two of these cases, the sensorimotor task depended on the severity of participants’ motor impairment or individual preference (Hohne et al., 2014, Tan et al., 2010), and another three combined MI and motor attempt to trigger neurofeedback (Carrere et al., 2021, Cincotti et al., 2008, Zhang et al., 2018). The remaining four studies consisted of two parts, with the BCI controlled initially through MI followed by motor attempt, or vice versa (Daly et al., 2009, Hortal et al., 2015, Norman et al., 2018, Pitt and Brumberg, 2022).

The upper limb(s) was most frequently studied (n = 116, 87%) with tasks mainly involving reaching (elbow extension-flexion) and/or grasping (wrist and finger extension-flexion). In the 15 paradigms involving the lower limbs, the tasks included imagining or attempting foot or ankle dorsiflexion.

In a proportion of studies (n = 11, 8%), participants were not instructed to perform or imagine movement, but rather were simply told to minimise movement and stay mentally focused. In one study, participants were asked to “find a mental condition” that successfully reduced alpha power, which in turn played a video, although the strategies employed by participants were not reported (Alves-Pinto et al., 2017). However, in another study, despite not being specifically directed to, it was reported that participants tended to use motor imagery to modulate SMR and control a visual cursor (Wolpaw & McFarland, 2004).

Two of the studies enrolling children employed MI of the upper limb (Bobrov et al., 2020, Jadavji et al., 2023), whilst the third used MI or motor attempt of the upper or lower limbs (Cincotti et al., 2008).

### 3.5 Feedback mode

Fig. 3B summarises the feedback modes used. The most prevalent mode was visual only (n = 34, 26%) and involved feedback on a display screen, for example, vertical or horizontal movement of a cursor towards a target, colour change of a target, or variations in bar height. Visual feedback was also incorporated in most interventions delivering multimodal feedback (n = 57, 43%). In 13 studies, visual feedback was delivered in the form of virtual reality, whereby participants’ sensorimotor EEG signals controlled the movement of virtual hands performing a motor task.

Haptic feedback was also commonly used, mostly delivered by a robotic device with the BCI using sensorimotor EEG signals to initiate movement. Robotic devices included exoskeletons, mechanical orthoses and continuous passive motion machines attached to participants’ impaired limbs. Other haptic feedback included vibrotactile (Vourvopoulos et al., 2019a) or brush (Hu et al., 2021) stimuli. A further mode of feedback involved functional electrical stimulation (FES) to facilitate movement. Robotic devices and FES were used solely (16 and 13 studies, respectively) or in combination with visual feedback on a display screen (23 and 15 studies, respectively).

Four studies administered electro-tactile stimulation to the tongue, along with visual and FES feedback (Remsik et al., 2018, Remsik et al., 2019, Sinha et al., 2021, Young et al., 2014). Other less common feedback modes included auditory and neuromuscular electrical stimulation.

Two of the paediatric studies delivered visual feedback on a display screen (Cincotti et al., 2008, Bobrov et al., 2020), whilst the other delivered both visual feedback and FES (Jadavji et al., 2023).

### 3.6 EEG-based sensorimotor neurofeedback parameters

This review focused on studies that report the use of sensorimotor EEG signals for neurofeedback. Where these details were provided, the frequencies and EEG channels used for neurofeedback, as well as the signal processing tools and features extracted, are listed for each study in Table 1. The extent to which these parameters were reported varied widely, often depending on the BCI design. Within a given study, the parameters used for online and offline EEG analysis were not necessarily the same. Therefore, parameters for online and offline EEG analysis, where performed, are listed separately. Overall, 72% (n = 96) of studies reported details of both online and offline parameters, although often details were incomplete (Table 1).

Most studies (n = 90, 68%) used a BCI that incorporated machine learning algorithms, such as Common Spatial Patterns (CSP) and Support Vector Machines, and/or classifiers, such as Bayesian or Linear, in their signal processing pipeline to identify participant-specific spatial and/or frequency features from the EEG signal for neurofeedback (Table 1). Twenty-five of these studies used unspecified classifiers and algorithms within the BCI2000 software system. Often, studies were focused on BCI system feasibility and used classification accuracy (CA) to evaluate the performance of the BCI model (*see* section 3.8.1*)*. The EEG parameters in these designs tended to comprise a broader frequency range (e.g., 5–30 Hz or 0.05–40 Hz), including the alpha and beta components of the sensorimotor rhythm. After calibration, this would then be refined to a participant-specific frequency band for training sessions. There was variability in the EEG channels used for neurofeedback: whilst some studies reported the specific channels used, such as C3, Cz and C4 in the sensorimotor region, often channels or spatial components were selected after calibration by classifiers that showed the strongest cortical activation or highest power associated with different mental states or tasks.

Across the studies that reported frequency bandwidths and resolution, there was wide variation in the selected parameters: 50% of studies (n = 66) reported a defined frequency range (e.g., alpha/mu 8–12 Hz), while 44% (n = 59) reported a broader range incorporating both alpha and beta frequencies (e.g., 8–40 Hz). Eight studies did not report the EEG frequencies used (Ang et al., 2010, Broetz et al., 2010, Caria et al., 2010, Cho et al., 2016, Mukaino et al., 2014, Ono et al., 2013, Varkuti et al., 2013, Young et al., 2014). Four studies delivered two distinct frequencies as neurofeedback, with alpha or beta as reward neurofeedback, and surrounding delta/theta/beta/gamma as inhibitory neurofeedback (Cho et al., 2015, Erickson-Davis et al., 2012, Lee et al., 2015, Rayegani et al., 2014). Another study delivered different neurofeedback frequencies depending on whether the task was performed with the affected or unaffected limb (Silvoni et al., 2013).

EEG features used for neurofeedback were mainly focused on detecting and processing ERD of mu, alpha, beta or an unspecified rhythm (n = 103, 77%). Five papers mention ERD in the introduction or results, but this was not specified within the methodology (Ang et al., 2014a, Ang et al., 2011, Broetz et al., 2010, Chen et al., 2021, Pfurtscheller et al., 2000). A further fourteen studies used SMR power, often without specifying the nature of the change, and two studies used functional connectivity in the alpha frequency band for EEG neurofeedback (Mottaz et al., 2018, Mottaz et al., 2015). The remaining studies did not report a specific EEG feature used for neurofeedback (n = 11, 8%).

All three paediatric studies used ERD as the neurofeedback feature. However, this was detected across varying channels and frequencies. Two studies used CSP and/or classifiers to detect ERD in a broad frequency range across many channels (Bobrov et al., 2020, Jadavji et al., 2023). The remaining study utilised a narrower frequency range (3–14 Hz) over a sub-set of 59 channels, using unspecified classifiers within the BCI2000 software system (Cincotti et al., 2008).

### 3.7 (Offline) EEG analysis

Of the 133 included studies, 96 (72%) performed offline EEG analysis, mostly focusing on spectral power measures such as ERD/event-related spectral perturbation – ERSP (n = 90, 68%). A subset of studies (n = 28, 21%) analysed other quantitative spectral measures such as entropy, EEG:EEG coherence and cortico-muscular coherence, with one study analysing and comparing a large array of EEG features (Erickson-Davis et al., 2012). More recently published studies have focused on a range of functional and effective connectivity measures such as coherence (generalised partial directed coherence, magnitude squared coherence and the imaginary component of coherence), direct transfer function, global and local efficiency, clustering coefficient, node strength, network density and phase slope index (Chang et al., 2023, Kern et al., 2023, Mottaz et al., 2018, Mottaz et al., 2015, Remsik et al., 2021, Yuan et al., 2021, Zhan et al., 2022, Zhang et al., 2023).

A small number of studies additionally analysed measures of brain symmetry and lateralisation. Three studies looked at the brain symmetry index (BSI) to capture differences in spectral power between the cerebral hemispheres (Ang et al., 2014a, Kumari et al., 2022, Zhang et al., 2018). Other studies analysed ERD-based lateralisation index to assess the strength and/or timing of ERD in different brain regions in relation to specific cognitive or motor functions (Braun et al., 2017, Jia et al., 2022, Kumari et al., 2022, Remsik et al., 2019, Vourvopoulos et al., 2019a, Vourvopoulos et al., 2019b).

Regarding the paediatric studies, two analysed ERD modulation (Bobrov et al., 2020, Cincotti et al., 2008), while the remaining study did not perform offline EEG analysis (Jadavji et al., 2023).

### 3.8 Outcome measures

Reported outcomes included measures of BCI system feasibility, BCI participant performance and clinical outcome scores. Usability was also documented.

#### 3.8.1 BCI system feasibility

BCI classification accuracy (CA) was used in 20 studies to measure the feasibility and performance of the BCI system itself. CA reflects the classifier’s ability to accurately detect and differentiate between different mental states and translate the participant’s brain activity into commands (Yuan & He, 2014). All 20 studies reported that CA was better than chance level (50%). Fifteen reported accuracy was greater than the recommended minimum accuracy level for a BCI system (70%) for at least one training session (Kübler et al., 2004).

CA may also be reported as a measure of participant performance (see below).

#### 3.8.2 BCI participant performance

**Fig. 4:**
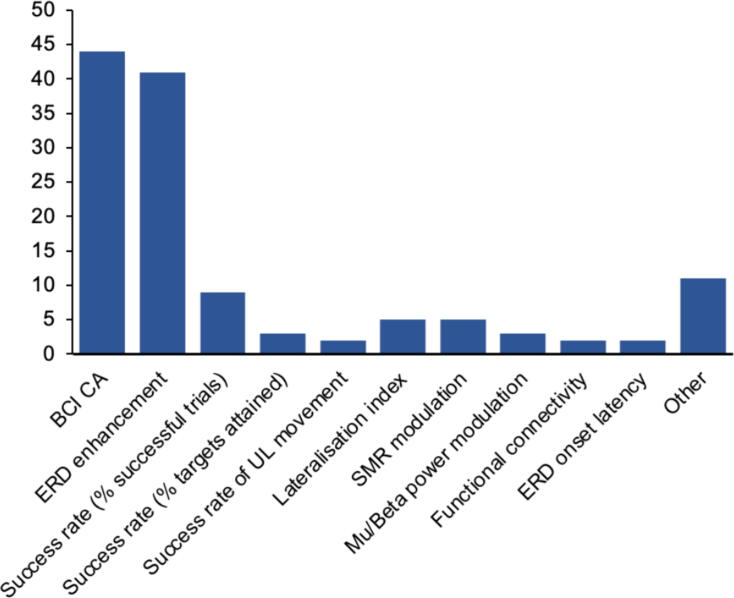
Number of studies that employed each BCI participant performance measure. BCI=Brain-Computer Interface, CA=Classification Accuracy, SMR=Sensorimotor Rhythm, UL=Upper Limb.

Just over half of the studies reported one or more measures related to participants’ performance in the BCI neurofeedback task (n = 73, 55%), as shown in Fig. 4. The enhancement of ERD from pre- to post- neurofeedback training – i.e., the performance measure most directly related to the targeted neurofeedback signal itself (ERD) – was reported in 41 articles (31%). However, 29 of these 41 articles displayed this result graphically without specifying numerical values in the text. A further stated there had been an enhancement of ERD but did not report values either graphically or in the text. For the nine studies that reported the value of ERD enhancement, this was reported differently across studies depending on their methodology. In some studies, ERD was expressed as the percentage change in power from baseline, (also described in some articles as the mu suppression score) while in others it was expressed as a ratio between the average band-power during the motor task and the reference period. Others reported the signed r-squared coefficient of determination value. In some articles, the pre- and post-intervention ERD values were reported; in others, only the change in ERD from pre- to post- intervention was stated and a few reported both. Some articles reported the group mean values of ERD (either percentage or ratio) for an experimental group versus a control group, or for the contralesional versus the ipsilesional hemisphere. The variability in reporting makes it difficult to compare values of ERD enhancement across studies.

For example, Chowdhury et al. (2020) calculated the ratio between the average band-power during the motor task and the reference period. They showed graphically that this measure decreased over the weekly neurofeedback sessions (i.e. there was a change in favour or mu ERD rather than ERS); they reported that the group-mean ratio changed from 1.03 to 0.74 over time, amounting to a change of −28.36%, which represents a statistically significant (p < 0.05) enhancement in ERD. In contrast Remisk et al. (2019) reported the signed r^2^ coefficient of determination value, calculated from the absolute mu power during movement trials compared with rest trials, with negative values indicating a mu ERD. They demonstrated a statistically significant enhancement in mu ERD for the ipsilesional hemisphere following therapy (mean r squared value pre- and post-intervention −0.142 and −0.161 respectively, p = 0.039).

CA was also used in 44 studies as a measure of how successfully participants controlled the BCI system (separately from reflecting system feasibility). This depicted the percentage of times participants successfully operated the BCI to trigger neurofeedback. CAs ranged from over 50 to above 90%, with accuracy tending to increase over intervention periods, demonstrating improved participant performance over time. Factors reported to have potentially influenced CA included fatigue (Prasad et al., 2010, Resquin et al., 2016), type of MI task (Pfurtscheller et al., 2000) and medication (Irimia et al., 2018). Other performance measures included success rate, defined as the percentage of successful trials or targets attained, and SMR modulation (not specified as ERD).

All three paediatric studies used BCI CA to measure performance. Changes in the neurofeedback feature itself, the mu ERD, were not reported.

#### 3.8.3 Clinical outcome scores

Most studies reported improvement in motor function using clinical outcome measures (n = 86, 65%), most commonly the Fugl-Meyer Assessment (FMA) (n = 59, 44%), Action Research Arm Test (n = 21, 16%) and/or (Modified) Ashworth Scale (n = 19, 14%). These measures reflect the large proportion of studies enrolling stroke participants. Twenty-two studies reported that improvements in clinical motor scores were sustained at follow-up, with 13 studies reporting statistically significant improvements. Follow-up time points ranged from one-to-twelve months post BCI therapy, with two studies reporting statistically significant improvement in upper limb FMA scores persisting at six (Zhang et al., 2023) and six-to-twelve (Biasiucci et al., 2018) months post-BCI training. For both of these studies, more BCI participants than controls achieved clinically significant scores.

However, most studies did not investigate the relationship between clinical outcomes and neurophysiological outcome measures. Twenty-eight studies (21%) explored an association between motor outcomes and participant performance. Twenty-one of these conducted a formal correlation analysis, with 15 studies reporting a statistically significant positive correlation (Table 1). The remaining seven performed informal correlation analysis i.e., associations were commented on, but no statistical analyses were reported.

Sixteen of the 28 studies explored an association between motor improvement and a specific ERD-related outcome, i.e., the performance measure directly related to the neurofeedback signal (ERD). Two of these were single case reports (Mukaino et al., 2014, Naros and Gharabaghi, 2015). The other 14 are described in Table 2. All 14 studies reported improvements in at least one clinical measure, with nine reporting statistically significant improvements (Table 2). Eight of these studies reported some improvement in clinical scales surpassing the minimal clinically important difference (MCID).

**Table 2:** A subset of included studies that explored clinical and ERD outcome measures. *ARAT*=Action Research Arm Test, *BCI*=Brain-Computer Interface, *EEG*=Electroencephalography, *ERD*=Event-Related Desynchronisation, *ERS*=Event-Related Synchronisation, *FMA*=Fugl-Meyer Assessment, *GS*=Grip Strength, *MCID=*Minimal Clinically Important Difference, *SMR*=Sensorimotor Rhythm, *UE*=Upper Extremity.

**Study Details**		**Design**	**Outcomes**		
Title	Author and Year Published	*N*	ERD outcome	Clinical motor outcome	Correlation
Contralesional Brain–Computer Interface Control of a Powered Exoskeleton for Motor Recovery in Chronic Stroke Survivors	Bundy et al., 2017	10	ERD enhancement displayed graphically but numerical values not specified in the text.	Statistically significant average increase of 6.2 in ARAT. Six out of 10 participants surpassed MCID. Significant improvements in secondary outcomes of GS, Motricity Index, and the Canadian Occupational Performance Measure.	Non-significant trend toward a positive relationship between ARAT score changes and ERD modulation per training run.
Longitudinal Analysis of Stroke Patients’ Brain Rhythms During an Intervention with a Brain-Computer Interface	Carino-Escobar et al., 2019	9	Alpha and beta ERD ‘trends’ across sessions displayed graphically but numerical values not specified in the text.	Three out of nine participants had improvements in FMA-UE of 3 scores or higher. Three patients improved by scores between 2 and 1. Three participants did not show improvements. Clinical and statistical significance not reported.	Linear predictive modelling showed significant relationship between alpha ERD enhancement and clinical recovery.
Longitudinal Electroencephalography Analysis in Subacute Stroke Patients During Intervention of Brain–Computer Interface with Exoskeleton Feedback	Chen et al., 2020	14 (7 in experimental group + 7 in control group)	ERD of channels C3 and C4 became significantly stronger post intervention. ERD enhancement displayed graphically but numerical values not specified in the text.	Statistically significant improvement for experimental and control group in FMA. Experimental group showed larger improvement than the control group (12.8 vs 7.1%). More patients obtained good motor recovery in the experimental group than did the control group (57.1% vs 28.6%). Four out of seven participants in experimental group surpassed MCID vs two participants in control group.	Participants with good recovery showed an enhanced ERD post intervention compared to pre intervention. Authors reported this as an implied correlation, but no formal analysis was conducted. Significance not reported.
EEG-Controlled Functional Electrical Stimulation Rehabilitation for Chronic Stroke: System Design and Clinical Application	Chen et al., 2021	32 (16 in experimental group + 16 in control group)	Significant mu and beta ERD enhancement across sessions displayed graphically but numerical values not specified in the text.	Significant improvements in FMA-UE and Kendall Manual Muscle Test in each group post intervention. Significantly higher improvements in FMA-UE and Kendall Manual Muscle Test in experimental group vs control group. Clinical significance not reported.	The change in laterality coefficient values based on mu ERD showed a high statistically significant positive correlation with the change in FMA-UE and Manual Muscle Test scores. The change in laterality coefficient values based on beta ERD showed a statistically significant positive correlation with change in FMA-UE.
Corticomuscular Co-Activation Based Hybrid Brain-Computer Interface for Motor Recovery Monitoring	Chowdhury et al., 2020	4	Overall trend of ERD enhancement for mu and beta bands. Statistically significant group-mean change in mu (−0.29; 28.36% reduction from baseline) and beta (−0.18, 17.20% reduction from baseline) ERD.	Statistically significant group mean improvements of 23.75 and 9.83 kg in ARAT and GS, respectively. Improvements in ARAT and GS surpassed MCID limits.	Significant correlations between mu/beta ERD and GS and ARAT at various EEG channel locations on the scalp.
Neurophysiological Substrates of Stroke Patients with Motor Imagery-Based Brain-Computer Interface Training	Li et al., 2013	14 (7 in experimental group + 7 in control group)	Significantly stronger ERD of unaffected sensorimotor cortex in experimental and control groups post intervention. Significantly stronger ERD of affected sensorimotor cortex for experimental group post training. ERD enhancement displayed graphically but numerical values not specified in the text.	Statistically significant improvements in FMA and ARAT scores for both groups. Statistically significant differences between groups observed post intervention in ARAT. No statistically significant differences between groups at different course periods in FMA. Clinical significance not reported.	Significant correlations between strength of ERD values over some brain regions and FMA and ARAT scores. Regression analyses showed significant relationships between ERD values of affected sensorimotor cortices and FMA and ARAT scores, and between ERD values of affected parietal lobe and ARAT scores.
Sensorimotor Rhythm-Brain Computer Interface with Audio-Cue, Motor Observation and Multisensory Feedback for Upper-Limb Stroke Rehabilitation: A Controlled Study	Li et al., 2022	24 (12 in experimental group + 12 in control group)	No significant change in mu ERD in bilateral hemisphere post intervention. Mu suppression values pre/post intervention: 1) Ipsilesional hemisphere 45.8 ± 28 (pre), 56.8 (47.9, 60.7) (post). 2) Contralesional hemisphere 62.4 (21.4, 72.9) (pre), 53.8 ± 26 (post). No significant difference between hemispheres.	Statistically significant improvements for both groups, but significantly higher improvements in FMA-UE and Wolf Motor Function Test post intervention for experimental vs control group. Post intervention, increase in FMA-UE and Wolf Motor Function Test surpassed MCID for all the patients in experimental group.	Non-significant trend between strength of mu ERD of contralesional or ipsilesional hemisphere and FMA or Wolf Motor Function Test.
A Multi-Target Motor Imagery Training Using Bimodal EEG-fMRI Neurofeedback: A Pilot Study in Chronic Stroke Patients	Lioi et al., 2020	4	ERD enhancement displayed graphically but numerical values not specified in the text.	Improvements in two out of four participants in FMA, with one participant improving by 6 (+31.5%; clinically significant) and the other by 3 (+6%; not clinically significant). Statistical significance not reported.	On a single case level, the authors noted an apparent association between ERD enhancement and FMA scores.
Brain-Controlled Functional Electrical Stimulation Therapy for Gait Rehabilitation after Stroke: A Safety Study	McCrimmon et al., 2014	9	Five participants exhibited significant increases in ERD/ERS. ERD enhancement displayed graphically but numerical values not specified in the text.	Improvements in five out of nine participants in gait speed, three participants in dorsiflexion active range of motion, five in the Six-Minute Walk test, and three in FMA. Two participants surpassed MCID in gait speed, and four in Six-Minute Walk test. Statistical significance not reported.	On a single case level, the authors noted five participants that exhibited motor improvement post training also exhibited a significant enhancement in ERD.
Hand Motor Rehabilitation of Patients with Stroke Using Physiologically Congruent Neurofeedback	Ono et al., 2018	9	Change in ERD in affected hemisphere from pre to post intervention was not statistically significant: 1) Experimental group 20 (−58, 27) (pre), 22 (17, 27) (post). 2) Control group 25 (15, 31) (pre), 21 (−3, 30) (post).	Statistically significant improvement in FMA and Modified Ashworth Scale post intervention period but not control period. Clinical significance not reported.	Non-significant correlation between ERD enhancement on the affected hemisphere and change in FMA post BCI training vs control period.
Applying a Brain-Computer Interface to Support Motor Imagery Practice in People with Stroke for Upper Limb Recovery: A Feasibility Study	Prasad et al., 2010	5	ERD/ERS change from the first to the last session was statistically significant for only two out of five participants. High degree of subject specificity in the evolution of ERD/ERS correlates over the course of BCI sessions.	Positive improvement in at least one measure was observed in all participants. Mean changes from baseline scores in Motricity Index (11.7%), ARAT (18%; two participants surpassed MCID), Nine Hole Peg Test (33.3%) and GS (20%). No mean improvements surpassed MCID. Statistical significance not explored.	Correlations were performed at single case level. Large correlation (r > 0.5) between at least one participant’s ERD/ERS ratio and an outcome measure score. The outcome measures scores of ARAT and GS had large correlation with ERD/ERS ratios of all the participants.
Ipsilesional Mu Rhythm Desynchronization and Changes in Motor Behavior Following Post Stroke BCI Intervention for Motor Rehabilitation.	Remsik et al., 2019	21	Significant decrease in mean mu at ipsilesional channel C4/C3 from pre (−0.142) to post (−0.161) intervention (expressed as the signed r^2^ coefficient of determination value, calculated from absolute power during movement trials compared with rest trials). Non-significant decrease in mean mu at contralesional channel C4/C3 from pre (−0.131) to post (−0.145) intervention. Non-significant effects in beta band.	Statistically significant improvement from baseline to post intervention and at one month follow-up in ARAT. Statistically significant improvement from baseline to post intervention but not at follow-up in GS. Statistically significant improvement from baseline to follow-up in Stroke Impact Scale. No significant results in secondary measures (including Stroke Impact scale, National Institutes of Health Stroke scale and Barthel scale). Clinical significance not reported.	Mu enhancement from baseline to post intervention in the ipsilesional hemisphere showed a non-statistically significant positive correlation with the change in ARAT scores.
Ipsilesional Mu Rhythm Desynchronization Correlates with Improvements in Affected Hand Grip Strength and Functional Connectivity in Sensorimotor Cortices Following BCI-FES Intervention for Upper Extremity in Stroke Survivors	Remsik et al., 2021	16	Largest, non-significant, increases in mu ERD for ipsilesional primary motor cortex and ipsilesional somatosensory association area. ERD enhancement displayed graphically but numerical values not specified in the text.	Mean improvements post intervention in hand GS (1.69 +/- 6.41) and ARAT (1.44 +/- 4.34). Clinical and statistical significance not reported.	Improved hand grip function showed a significant positive correlated with increased mu ERD from pre to post intervention in the ipsilesional primary motor cortex.
Applying Action Observation During a Brain-Computer Interface on Upper Limb Recovery in Chronic Stroke Patients	Rungsirisilp et al., 2023	17 (9 in experimental group + 8 in control group)	Significantly stronger ERD of channels C3/C4 in alpha and beta bands, and greater enhancement over time, in experimental group vs control group. Mean percentage ERD/ERS of C3/C4: 1) Experimental group: −30.8 +/- 12.96 (alpha), −26.3 +/- 7.39 (beta). 2) Control group −17.47 +/- 13.4 (alpha), −14.7 +/- 8.79 (beta).	Statistically significant improvement in FMA-UE for experimental group (5.67 +/- 3.09; surpassing MCID) vs control group (2.75 +/- 1.56; not surpassing MCID).	Significant correlation between change in FMA and strength of ERD of C3/C4 in the beta band. No significant correlation for alpha band.

Eight out of the 14 studies reported ERD enhancement from pre- to post-neurofeedback training. However, ERD enhancement was often shown graphically without numerical values noted in the text. The remaining six studies correlated changes in motor function with a single ERD measurement, rather than ERD modulation from pre to post training. Overall, six studies found a statistically significant correlation between motor improvements and an ERD-related outcome.

Thirteen studies conducted formal correlation analyses (and one informal) between motor improvement and additional performance measures, such as CA (n = 7) or coherence (n = 4). While these correlations are listed in Table 1, further details have not been examined in Table 2 as these outcomes are indirectly related to the neurofeedback signal. As these are indirect measures of neurofeedback performance, it is not possible to conclude that any observed motor improvements are attributable specifically to the neurofeedback training.

Only one paediatric study measured motor improvement clinically, reporting significant improvements in sub-scales of FMA, ARAT and Jebsen–Taylor function tests (Bobrov et al., 2020). However, associations between these improvements and neurofeedback performance were not explored.

#### 3.8.4 Usability

Thirty-one articles commented on the usability of the BCI neurofeedback system. Standard usability assessment tools included NASA task load index (Kumari et al., 2022, Sakamaki et al., 2022, Zulauf-Czaja et al., 2021), visual analogue scale (Chowdhury et al., 2018, Prasad et al., 2010, Zich et al., 2017), simulator sickness questionnaire (Vourvopoulos et al., 2019b, Vourvopoulos et al., 2019c), Quebec user evaluation of satisfaction with assistive technology (Nishimoto et al., 2018, Zulauf-Czaja et al., 2021) and system usability scale (de Castro-Cros et al., 2020). Other studies did not report use of standard scales but conducted questionnaires or interviews exploring participants’ levels of enjoyment and satisfaction, motivation and fatigue, workload (mental or physical demand) and comfort. No studies reported adverse events. Generally, participants reported enjoyment and satisfaction with the EEG-BCI training.

Eight studies reported increases in fatigue over the course of BCI training, two of which suggested that fatigue might have contributed to a larger variability or decline in BCI performance (Prasad et al., 2010, Resquin et al., 2016). Four studies delivered one or two training sessions (Hortal et al., 2015, Jadavji et al., 2023, Resquin et al., 2016, Sakamaki et al., 2022), whilst three delivered 10 – 12 sessions (Frolov et al., 2017, Frolov et al., 2016, Prasad et al., 2010). The final study required participants to undertake at least 60 training sessions at home over 12 weeks (Bundy et al., 2017). This subset of studies reflects the broader variability across all identified studies with respect to the number of runs, trials, sessions and rest intervals employed in training interventions.

To improve engagement in the EEG-BCI systems, participants’ suggestions included a “pause” feature to reduce fatigue (Leeb et al., 2013), introducing variations in the neurofeedback game’s animations and auditory stimuli (de Castro-Cros et al., 2020), and increasing the challenge level (Prasad et al., 2010).

Two of the paediatric studies reported usability. One study administered a questionnaire assessing BCI system acceptance, with the children reporting independent use of the system (Cincotti et al., 2008). The other paediatric study explored levels of fatigue, comfort and engagement (Jadavji et al., 2023). The most common complaints were headset discomfort (58%) and muscle fatigue (50%). The children ranked the BCI intervention as comparable to a long car ride.

### 3.9 Control and comparison groups

Overall, 63 studies incorporated a control condition. Most enrolled a distinct control group using a between-participant design (n = 51, 38%). Of these, 34 studies recruited only participants with neurological motor impairments who were randomly assigned to an experimental or control group. In the experimental group, the BCI system delivered neurofeedback based on patients’ EEG signals (EEG-BCI), whereas the control group received sham feedback, or training that did not involve the BCI system. The remaining 17 between-participant study designs enrolled healthy volunteers as the control group, and both patients and controls received the same experimental intervention (EEG-BCI).

A smaller proportion of studies carried out a within-participant design with each patient serving as their own control (n = 12, 9%). In some, patients engaged in a cross-over control design where outcomes were measured during a control versus a BCI therapy phase (Mottaz et al., 2018, Remsik et al., 2018, Remsik et al., 2021). Alternatively, patients received EEG signal-driven feedback versus sham feedback (Alves-Pinto et al., 2017, Mukaino et al., 2014, Ono et al., 2013, Ono et al., 2018, Takahashi et al., 2012, Tsuchimoto et al., 2019, Wada et al., 2019), or engaged in trials with versus without neurofeedback (Kasahara et al., 2018, Pfurtscheller et al., 2000). Five studies compared the clinical improvement in patients’ motor functioning between trial types or design phases (Mukaino et al., 2014, Ono et al., 2013, Ono et al., 2018, Takahashi et al., 2012, Wada et al., 2019). Three of these reported greater improvement after EEG-driven feedback compared to sham feedback (Mukaino et al., 2014, Ono et al., 2013, Takahashi et al., 2012), and two reported improvements only after the BCI therapy phase compared to the control phase (Ono et al., 2018, Wada et al., 2019). However, only one of these reported an associated trend between improvement in motor function and increase in ERD (neurofeedback performance) (Ono et al., 2018).

Eighteen studies compared two or more techniques of delivering neurofeedback. For example, three studies compared two neurofeedback modes: visual feedback on a display and haptic feedback via a robotic device (Frisoli et al., 2012, Ono et al., 2014, Sakamaki et al., 2022). Two studies reported that the BCI CA of the robotic feedback condition matched that of the visual feedback condition (Frisoli et al., 2012, Sakamaki et al., 2022). The third study reported improvement in finger function in the robotic feedback condition only (Ono et al., 2014).

Control or comparison groups were not included in the paediatric studies.

### 3.10 Cognitive strategies and additional therapies

#### 3.10.1 Motor imagery strategies

Some studies indicated that BCI performance was influenced by the type of MI strategy performed. For example, a single case study reported that the participant’s CA varied between 50 and almost 100%, with right- and left-hand MI strategy yielding relatively moderate classification rates, whilst foot MI increased CA considerably (Pfurtscheller et al., 2000). Additional studies suggested the importance of identifying participant-specific MI strategies that optimally support BCI control (Leeb et al., 2013, Neuper et al., 2003). In a further study in which participants explored different strategies, participants reported that employing more complex MI strategies (imagining hair combing and ironing vs opening/closing of hand) was more effective at controlling the BCI (Lioi et al., 2020).

#### 3.10.2 Augmentative cognitive strategies

Cognitive strategies beyond MI were proposed to participants to assist them in controlling the BCI and to augment the neurofeedback in two studies: in one study, researchers suggested participants try mentally counting numbers (Spychala et al., 2020). In another study, to control the BCI, participants were asked to attempt and subsequently imagine ‘finger individuation’ (i.e., extending one finger while inhibiting the movement of another) (Norman et al., 2018). This task required complex cognitive effort to make cue-based decisions.

#### 3.10.3 Additional therapies

Some studies (n = 28, 21%) incorporated therapies in addition to BCI training such as physiotherapy, occupational therapy, or conventional rehabilitation therapy. Conventional treatments included electrical stimulation (Chen et al., 2020, Li et al., 2022), Activities of Daily Living training (Biasiucci et al., 2018, Li et al., 2022) and acupuncture therapy (Li et al., 2013). Other studies conducted action observation (n = 7) (Choi et al., 2020, Kumari et al., 2022, Ono et al., 2018, Rungsirisilp et al., 2023, Spychala et al., 2020, Wada et al., 2019, Wang et al., 2018) or digital mirror box training (n = 2) (Ono et al., 2018, Wada et al., 2019).

Cognitive strategies and additional therapies were not explored in the paediatric studies.

## 4 Discussion

Sensorimotor EEG-based neurofeedback has exciting therapeutic potential as an intervention for under-served clinical populations such as childhood-onset movement disorders. This scoping review maps the breadth of research exploring EEG-based sensorimotor neurofeedback in both children and adults with neurological motor impairment. The temporal profile of included articles (Fig. 2A) indicates the rapid expansion of the field, with growing interest from engineers and clinicians in the potential benefits for patients. However, there is a paucity of evidence on the application of these systems in children, with most studies focusing on adults with stroke (Fig. 2B). Furthermore, work is largely at an early stage on the spectrum from physiological proof-of-principle to full clinical translation, and this is reflected by the OCEBM classification of studies, with the majority being level 4. Even among articles described as RCTs, most were unpowered studies without sample size estimations, thus cannot be classified as level 2 evidence. Rather, these are exploratory pilot studies. This is to be expected given this is a relatively new and emerging field, and we emphasise the importance of these early studies for answering important methodological questions which will inform the design of full-scale RCTs in due course.

### 4.1 EEG-based sensorimotor neurofeedback

This review focused on studies using EEG-based sensorimotor neurofeedback. If real-time EEG data are to be used as the basis of a proposed clinical neurorehabilitation intervention, then both the recording parameters used, and the data quality are of paramount importance. However, there was considerable variability across the literature in how EEG parameters were reported. Nine studies were excluded from the review at the full-text screening stage as the reported EEG details were insufficient to determine the nature of the signal being used for neurofeedback. For example, some papers stated that the BCI system “recognised the brain signals of patients and converted these into motor commands” but did not specify further methodological details. Across the included studies, there was also considerable variation in EEG frequency ranges, topography and the use of processing algorithms and classifiers (Table 1). To aid clarity, we separated the parameters reported in each paper into those relating to the online EEG used for neurofeedback and those relating to subsequent offline analysis.

Online signal processing involves extracting immediate, relevant features from EEG, as it is being recorded, to be used as neurofeedback. Almost half of the studies reported online EEG parameters covering multiple frequency bands, e.g., 0–45 Hz. Within this group, a small proportion used signal processing techniques to determine optimal participant-specific frequency ranges within the broader spectrum, but most did not. Therefore, there is ambiguity as to which frequency/rhythm may be influencing any neural effects observed.

Offline signal processing can be employed following calibration sessions of BCI training, or after the neurofeedback intervention itself, to assess participant performance and to perform a more comprehensive evaluation of changes in brain activity. This can include identifying and extracting complex patterns and nuanced EEG features related to specific cognitive states, tasks, or conditions (Mrachacz-Kersting & Aliakbaryhosseinabadi, 2018). Where offline EEG analysis was reported, frequency ranges were sometimes specified in more detail, but still not consistently.

Accuracy and timing of neurofeedback are further key considerations. Examples of raw EEG data were rarely provided, or such figures were often too small for readers to judge the data quality or exclude the possibility of significant contamination by EMG or other artefacts. When providing a participant with “real-time” feedback of their EEG activity, there is necessarily a lag-time between the detection of EEG signal change and the delivery of feedback. This is because a real-time system needs to record and process “packets” of data of a given length, which will vary between systems and studies, as will the interval at which feedback to the participant is updated (the feedback update interval (Darvishi et al., 2017)). Given the speed of physiological neural activity, shorter neurofeedback update intervals are likely to facilitate individuals in learning to modulate their EEG signals effectively. In the context of gaming and visual feedback, a “lag” is felt with an update rate of more than around 200 ms.

In practice, online processing, with appropriate decision-making and adjustments, can be challenging to implement and the ideal of “instantaneous” neurofeedback is difficult to achieve. Of the studies that reported the feedback update interval, these ranged from four milliseconds to one second, whereas others did not describe these temporal details, or reported that “data were transmitted in real-time” or that feedback was “sufficiently fast”.

### 4.2 Performance and outcomes

There was also considerable variability across the literature in the performance measures and outcomes reported. These included BCI system performance, BCI participant performance, participant improvement in the selected aspect of brain activity (e.g., mu ERD) during the study, changes in other measures of brain function (e.g., neuronal connectivity), clinical outcome scores aiming to detect changes in motor function and, finally, correlation between the above measures.

We highlight the distinction between the performance of the BCI system itself and the performance of the participant: BCI system performance is a measure of the technical performance and effectiveness of the interface in correctly detecting and classifying the relevant changes in cortical activity, often expressed as classification accuracy (*see* section 3.8.1). It does not directly translate into, or guarantee, participant proficiency in controlling a BCI. Conversely, participant performance is a measure of the ability of an individual to learn effective control of a BCI and is influenced by many other factors such as session design, participant adaptation, feedback mechanisms, ability to perform motor imagery, fatigue and cognitive factors. It can be difficult to separate these aspects, due to the interaction between the BCI and participant, but it is important that they are considered. In addition, neurofeedback studies using BCIs for neurorehabilitation will often aim to enhance a particular feature of brain activity (e.g., mu ERD). Therefore, it is relevant to report not only whether the participants could use/control the BCI but also whether participants showed an *enhancement* of this brain activity feature between the start and end of the study (or pre- and post-training), indicating neuroplasticity.

The outcomes and performance measures reported often reflected the nature of the paper and whether the focus was on the development of an assistive/restorative BCI for those lacking movement or on developing/testing a BCI to provide neurofeedback for sensorimotor training (rehabilitative BCIs). Again, there is a degree of overlap: even where BCIs are used primarily with an assistive/restorative purpose, there will effectively be an element of positive feedback to the participant through the successful performance of an action (e.g., movement of a wheelchair or a remote-controlled toy car), which in turn may lead to neuroplastic change. Since the defined question for our review related specifically to how neurofeedback has been used in rehabilitation, papers reporting the development of assistive BCIs without a particular focus on neurofeedback were excluded. However, even some of the included papers, which focused on rehabilitation, measured performance purely based on the ability of the BCI system to detect the relevant change in cortical activity, rather than reporting participant performance separately *(see* section 3.8.1*)*.

The variability in methodology and reporting of ERD enhancement makes it difficult to compare findings across studies. The two specific articles mentioned as examples in section 3.8.2 both provide detailed descriptions of their methodology and analysis, and both demonstrate statistically significant enhancement in ERD following the intervention, but it remains difficult to compare the findings. A consideration for future groups reporting EEG-based neurofeedback studies would be to include a measure of the effect size, which may facilitate comparison across studies to some extent. Open source sharing of methodologies and raw data could also be beneficial.

While most studies reported participant performance in the neurofeedback task and reported improvement in motor function in terms of a clinical outcome score, fewer papers investigated whether the reported motor/clinical improvement correlated with the feedback-related neural changes. This highlights a very important knowledge gap: without this form of study design and analysis, it is not possible to determine whether improved clinical scores arise specifically from the neurofeedback training, or whether they could just reflect a non-specific improvement relating to engagement in a study and its associated motor activities. Demonstration of “brain-behaviour relationships” is therefore a crucial step in building an evidence-base for neurofeedback interventions (Khademi et al., 2022, Mottaz et al., 2018, Ros et al., 2020).

Although many studies included in our review focus on ERD enhancement, others explored additional EEG outcome measures that may evolve our understanding of the impact of neurofeedback training. These features include measures of spectral power distribution such as the brain symmetry index or lateralisation index, measures of communication across brain regions, such as coherence and directed transfer function and network analysis parameters such as the global efficiency and clustering coefficient (see Section 3.7). Exploring these phenomena provides valuable insights into how neurofeedback training can modulate not only the original target feature (i.e., ERD), but also the complex and dynamic networks within the brain. Many motor disorders, including dystonia, are now considered network disorders (Latorre et al., 2020, McClelland and Lin, 2023), so exploring the relationship between these different neurophysiological phenomena is pertinent to understanding their pathophysiology. Although not a focus of the current review, studying the response to neurofeedback in healthy volunteers can also reveal important insights into plasticity within these sensorimotor networks and how this in turn relates to motor function/behaviour. Comparing how these networks function differently between participants with and without movement disorders could in turn inform the development of more effective interventions.

### 4.3 Clinical population

Despite the growing interest in this field, there is a significant gap in research investigating EEG-based neurofeedback for rehabilitation in patients with neurological motor impairments other than adult-onset stroke (Fig. 2B). In particular there are very few studies in children, with only three paediatric studies meeting the criteria for this review (Bobrov et al., 2020, Cincotti et al., 2008, Jadavji et al., 2023).

This is concordant with findings from a recent scoping review, published since our original search, which focussed on improved motor outcomes in children and adults with non-progressive neurological disorders undergoing BCI-based neurofeedback training (Behboodi et al., 2022). Although aiming to explore the scope of the published literature in adults and children, all 23 of their included studies were in adults, with 22 in stroke and one involving adults with incomplete spinal cord injury (Behboodi et al., 2022). Their review excluded progressive neurological conditions such as Parkinson’s Disease and Amyotrophic Lateral Sclerosis and only included studies in which participants attempted a voluntary motor task; paradigms exploring MI-induced mu modulation were excluded. Although for such reasons the Behboodi review is distinct from ours, it is notable that the authors aimed to map research in both children and adults but ultimately only included adult studies. This reinforces our finding that BCI research in children with neurological motor impairments is sparse.

Whilst we were keen to explore the literature on EEG-based neurofeedback across a broader range of neurological motor conditions, articles focussing on non-stroke diagnoses amounted to only 30 articles in total (23%). By including the literature on stroke our approach allowed us to capture the most extensive experience in the EEG-based neurofeedback literature. Potential reasons for the strong emphasis on adult stroke in the BCI-neurofeedback field are likely to be its high prevalence and a high level of motivation for research participation among individuals with stroke. The individuals enrolled in these studies would be expected to have had normal neurological development prior to their adult-onset stroke and so are likely to have established typical patterns of movement and sensorimotor neuronal circuitry. There is still likely to be considerable heterogeneity, but tasks such as motor imagery may therefore be more straightforward to convey to participants, and data interpretation may be less confounded by variables relating to developmental cortical re-organisation, than in participants with perinatal or childhood-onset disorders. A clear understanding of the effects of neurofeedback in conditions such as stroke is therefore very informative and some of the principles are likely to be relevant when considering neurofeedback interventions in general. Nevertheless, it is important that comprehensive studies are also conducted in children and individuals with other adult-onset neurological disorders, all of which are currently under-represented in research (Fig. 2B).

#### 4.3.1 Challenges of paediatric studies

Challenges of enrolling children in BCI/neurofeedback research include their diverse aetiologies, complex medical needs, cognitive and communication impairments, hyperkinesis, and a lack of paediatric-appropriate equipment (such as smaller commercial headsets) and engaging feedback systems (Jadavji et al., 2023), (see also section 3.8.4). Despite these challenges and the rigorous regulations applicable to research in children, it is important that dedicated paediatric neurophysiological studies are conducted and that the development of new therapies for children is not simply based on extrapolation from adult studies. Accepted models of “normal” sensorimotor neurophysiology may not be applicable when a brain injury has occurred early in development, as is the case in cerebral palsy and in some genetic conditions (McClelland, 2017). Given the potential for cortical reorganisation following early brain injury, one cannot presume that cortical sensorimotor processing will occur in the expected brain regions, or what typical neurofeedback paradigms might produce in such complex brains (Basu et al., 2010, Eyre et al., 2001, Staudt et al., 2002). It should not be forgotten that this is also a consideration in adults with cerebral palsy. Additionally, neuroplasticity is generally greater during childhood than in adulthood and the underlying neurological substrates of plasticity vary throughout development (Ismail et al., 2017, Tien and Kerschensteiner, 2018). As a result, an insult to the brain in childhood may have different consequences from an insult during adulthood (McClelland and Lin, 2021, McClelland et al., 2021). Likewise, the effects of an intervention may also have different consequences in childhood compared with adulthood. Indeed, there is evidence that children could be good candidates for EEG-BCI interventions due to their neuroplasticity (Jadavji et al., 2022, Jadavji et al., 2021, Zhang et al., 2019). For example, in one study, 12 children were able to perform mental strategies (MI and goal-oriented) to control a toy car and computer cursor (Jadavji et al., 2021) and in another study, eight children with quadriplegic CP controlled a powered wheelchair via EEG activity (Floreani et al., 2022). (These studies did not meet inclusion criteria for the formal review as they did not involve specific sensorimotor EEG-based neurofeedback, but the findings are pertinent when considering BCI research in children).

The three paediatric studies included in our scoping review (Bobrov et al., 2020, Cincotti et al., 2008, Jadavji et al., 2023) enrolled children with motor impairment diagnoses limited to CP (hemiplegic, spastic diplegic, tetraplegic and quadriplegic), Spinal Muscular Atrophy II and Duchenne Muscular Dystrophy. Dyskinetic/dystonic CP or other childhood-onset movement disorders were not represented, highlighting the need for research to assess the feasibility of EEG-BCI systems in this population.

### 4.4 Cognitive strategies

Our secondary question related to strategies applied to augment the effects of neurofeedback.

Although a large proportion of the included studies asked participants to perform MI to control the BCI, only two studies actively employed additional cognitive strategies to augment the neurofeedback (e.g., counting numbers), and the impact of these strategies was not explored in detail (Norman et al., 2018, Spychala et al., 2020). However, it is important to acknowledge that participants are likely to try various strategies of their own accord. For example, in two articles, participants were asked to retrospectively describe any strategies they used to control the BCI, and which appeared most effective, although this was not systematically tested (Lioi et al., 2020, Vourvopoulos et al., 2019b). One of these studies commented that their participants reported trying different strategies to control the BCI on different days, which could have influenced variability across sessions in BCI performance and in the resulting behavioural and neural changes (Ros et al., 2020, Vourvopoulos et al., 2019b).

It is acknowledged that sensorimotor paradigms may be more difficult to conduct in patients with childhood-onset motor impairments who have faced limitations in performing motor tasks throughout their lives. Indeed, physiological correlates of motor imagery are reduced in spastic CP (Jongsma et al., 2016), Parkinson’s Disease (Tremblay et al., 2008) and in focal hand dystonia (Quartarone et al., 2005). Nevertheless, the observation that MI is used as a strategy by individuals with Parkinson’s Disease (Nonnekes et al., 2019) and by children with dystonia (Butchereit et al., 2022), indicates its potential for improving motor performance. However, it is difficult to understand the exact nature of the MI in these individuals and whether it is used similarly across participants. The most effective strategies may vary between individuals and across different age-groups, necessitating a personalised approach (Floreani et al., 2022). Thus, more research is required to better understand the physiological correlates of this phenomenon in this population, along with a comprehensive evaluation of the role of cognitive strategies and their potential to augment EEG-BCI performance in both adults and children with neurological motor impairments.

### 4.5 Limitations and recommendations for future research

The methodology of a scoping review inherently limits researchers to mapping literature without providing analytical interpretation. However, even when researchers aim to conduct a systematic review, the limited breadth of adequately reported evidence hinders critical analysis, often making a scoping review more appropriate. To overcome this barrier to critical analysis for future work in this field, greater clarity of reporting is required regarding the raw neurophysiological data processed by BCI systems, along with greater transparency regarding the actual neurofeedback paradigms. Many commercial systems, such as BCI2000, employ various algorithms and classifiers for signal acquisition, signal processing, and neurofeedback, and the specific processes are unclear. Additionally, studies using Emotiv may lack clarity regarding the exact topography from which EEG signals are detected.

In the interests of transparency and the ability to reproduce published work, we advocate that all online neurofeedback parameters, including feedback update rate, should be reported, along with details of both the online and offline analyses. Where neurofeedback aims to enhance a particular neurophysiological feature (such as mu ERD), the resulting degree of ERD enhancement should be documented, and the effect size reported. Without such details it is difficult to understand which parameters are effective in providing neurofeedback for different patient populations, limiting the depth of insight that can be derived from these studies. Additionally, we highlight the importance of understanding the brain-behaviour relationship and recommend that authors investigate whether reported improvements in motor performance or clinical outcome scores correlate with the enhancement of the neurophysiological parameters being studied (Table 2).

A key finding of this scoping review was the lack of detailed reporting across many studies. However, the exercise identified several studies that adhered to good reporting practice. We define good practice as providing sufficient detail to enable the complete replication of a study’s methodology. Examples include, but are not limited to, studies by (Kumari et al., 2022, Pichiorri et al., 2015, Vourvopoulos et al., 2019a, Vourvopoulos et al., 2019b, Li et al., 2022, and Wada et al., 2019).

The CRED-nf checklist recommends guidelines for the design and reporting of clinical and cognitive-behavioural neurofeedback studies (Ros et al., 2020), including details of the online and offline EEG analyses. The potential role of strategies and the consideration of neurofeedback-specific versus non-specific factors is also highlighted (Ros et al., 2020). Although not specifically developed for studies of sensorimotor feedback in neurological motor impairment, the underlying principles and the checklist are equally applicable to studies in this field.

We also recommend that future research into EEG-based neurofeedback training for patients with neurological motor impairments be designed and conducted by multidisciplinary teams. The combined expertise of both engineers and clinical neurophysiologists is invaluable in creating robust study designs. Such collaboration will enhance the reporting standards of EEG neurofeedback parameters and analyses, which in turn is fundamental to investigating the relationship between neurofeedback training-induced EEG changes and observed clinical motor improvements.

## 5 Conclusion

There has been a rapid growth of interest in innovative EEG-based neurofeedback techniques. However, this review highlights that much of the work is still exploratory and that greater transparency is required in reporting of EEG parameters. Although several neurofeedback studies have reported improved clinical outcomes in adult stroke patients, very few have provided evidence that these outcomes relate specifically to the EEG-based neurofeedback (Table 2), limiting the conclusions that can be drawn. Furthermore, the reporting of neurophysiological parameters is often insufficient to allow reproducibility of the methodology. Whilst evidence of improved clinical outcome is the ultimate goal for neurofeedback studies, we consider that there first needs to be robust documentation of the neurophysiological methods being applied, both for the online and offline data acquisition and analysis. This requires a comprehensive and systematic approach, evaluating parameters across individuals of different ages and in different patient groups. It is also critical to assess the relationship between changes in brain activity triggered by the neurofeedback and any observed clinical improvement. Finally, there is a huge gap regarding the role of EEG-based neurofeedback in children with movement disorders. Pioneering work on the use of BCIs by children has shown promising results (Floreani et al., 2022, Jadavji et al., 2022, Zhang et al., 2019) but the specific role of sensorimotor neurofeedback in children with dystonia and dystonic/dyskinetic CP is relatively unexplored. Understanding the potential benefits and challenges of implementing EEG-based BCIs in paediatric cohorts holds significant promise for advancing neurorehabilitation strategies tailored to children with dystonia and dystonic/dyskinetic CP.

## Conflict of interest statement

Dr. Adam Kirton is co-cofounder and CMO of Possibility Neurotechnologies, a pre-revenue start-up company designing personalized BCI solutions to enable children with severe neurological disabilities. He holds a minority equity position but receives no income or other compensation from the company.

## CRediT authorship contribution statement

**Elena Cioffi:** Conceptualization, Methodology, Investigation, Formal analysis, Data curation, Writing – original draft, Writing – review & editing, Visualization. **Anna Hutber:** Investigation, Formal analysis, Data curation, Writing – original draft, Writing – review & editing. **Rob Molloy:** Investigation, Validation, Writing – review & editing. **Sarah Murden:** Investigation, Validation, Writing – review & editing. **Aaron Yurkewich:** Writing – review & editing. **Adam Kirton:** Writing – review & editing. **Jean-Pierre Lin:** Writing – review & editing. **Hortensia Gimeno:** Conceptualization, Methodology, Validation, Writing – review & editing, Supervision, Funding acquisition. **Verity M. McClelland:** Conceptualization, Methodology, Investigation, Validation, Writing – original draft, Writing – review & editing, Supervision, Funding acquisition.
