# Supplementary material for: EEG-based sensorimotor neurofeedback for motor neurorehabilitation in children and adults: A scoping review
Source: Clin Neurophysiol. 2024 Nov;167:143–66. doi: 10.1016/j.clinph.2024.08.009 (PMC11845253; doi:10.1016/j.clinph.2024.08.009)
Supplement: Supplementary Data 1 [file mmc1.docx]

**Supplementary Material**
Appendix A – Search Strategy for Medline (Ovid)

| **Concept 1: Neurofeedback** | **Concept 2: Augmented strategies and approaches** | **Concept 3: Motor impairment** |
| --- | --- | --- |
| Neurofeedback [MeSH]  OR neurofeedback [Text Word]  OR Feedback, Sensory [MeSH]  OR Biofeedback, Psychology [MeSH]  OR biofeedback [Text Word]  OR alpha feedback [Text Word]  OR alpha neurofeedback [Text Word]  OR alpha biofeedback [Text Word]  OR brainwave feedback [Text Word]  OR brainwave neurofeedback [Text Word]  OR alpha brainwave feedback [Text Word] | Cognitive strateg* [Text Word]  OR compensatory strateg* [Text Word]  OR CO-OP [Text Word]  OR Cognitive orientation to daily occupational performance [Text Word] | Movement Disorders [MeSH]  OR movement disorder* [Text Word]  OR Motor Skills Disorders [MeSH]  OR motor skills disorder* [Text Word]  OR motor deficit* [Text Word]  OR motor impairment* [Text Word]  OR developmental coordination disorder* [Text Word]  OR neurological disorder* [Text Word]  OR Dyskinesias [MeSH]  OR dyskinesia* [Text Word] |
| OR electroencephalography feedback [Text Word]  OR electroencephalography neurofeedback [Text Word]  OR electroencephalography biofeedback [Text Word]  OR electroencephalography alpha biofeedback [Text Word]  OR electroencephalography alpha neurofeedback [Text Word]  OR electroencephalography alpha feedback [Text Word]  OR alpha electroencephalography feedback [Text Word]  OR EEG feedback [Text Word]  OR EEG neurofeedback [Text Word]  OR EEG biofeedback [Text Word]  OR EEG alpha biofeedback [Text Word]  OR EEG alpha neurofeedback [Text Word]  OR EEG alpha feedback [Text Word]  OR alpha EEG feedback [Text Word] | OR occupational therapy [MeSH]  OR occupational therap* [Text Word]  OR ergotherap* [Text Word] | OR Dystonia [MeSH]  OR dystoni* [Text Word]  OR diurnal dystoni* [Text Word]  OR limb dystoni* [Text Word]  OR muscle dystoni* [Text Word]  OR Cerebral Palsy [MeSH]  OR cerebral palsy [Text Word]  OR Parkinson Disease [MeSH]  Or Parkinson disease [Text Word]  OR Huntington Disease [MeSH]  OR huntington disease [Text Word]  OR Stroke [MeSH]  OR stroke [Text Word]  OR Multiple Sclerosis [MeSH]  OR multiple sclerosis [Text Word] |
| OR Brain-Computer Interfaces [MeSH]  OR brain-computer interface* [Text Word]  OR brain machine interface* [Text Word] | OR physical therapy modalities [MeSH]  OR physical therap* [Text Word]  OR physiotherap* [Text Word]  OR neurophysiotherap* [Text Word]  OR neurological physiotherap*[Text Word] |  |
| OR electromyography feedback [Text Word]  OR electromyography neurofeedback [Text Word]  OR electromyography biofeedback [Text Word]  OR EMG feedback [Text Word]  OR EMG neurofeedback [Text Word]  OR EMG biofeedback [Text Word] |  |  |
| Sensorimotor rhythm feedback [Text Word]  Sensorimotor rhythm neurofeedback [Text Word]  Sensorimotor rhythm biofeedback [Text Word]  Sensory motor rhythm neurofeedback [Text Word]  Sensory motor rhythm feedback [Text Word]  Sensory motor rhythm biofeedback [Text Word]  SMR feedback [Text Word]  SMR neurofeedback [Text Word]  SMR biofeedback [Text Word] |  |  |
